# A simple and reproducible breast cancer prognostic test

**DOI:** 10.1186/1471-2164-14-336

**Published:** 2013-05-17

**Authors:** Luigi Marchionni, Bahman Afsari, Donald Geman, Jeffrey T Leek

**Affiliations:** 1 The Sidney Kimmel Comprehensive Cancer Center, Johns Hopkins University School of Medicine, 1550 Orleans Street, Baltimore, MD 21231, USA; 2 Department of Biostatistics, Johns Hopkins Bloomberg School of Public Health, 615 North Wolfe Street, Baltimore, MD 21205, USA; 3 Institute for Computational Medicine, Johns Hopkins University, 3400 North Charles Street, Baltimore, MD 21218, USA; 4 Department of Applied Mathematics and Statistics, Johns Hopkins University, 3400 North Charles Street, Baltimore, MD 21218, USA; 5 Center for Computational Biology, Johns Hopkins University, Baltimore, MD 21205, USA

**Keywords:** Reproducible research, Gene expression analysis, Biomarkers, Top scoring pair, Prediction, Genomics, Personalized medicine, Breast cancer, MammaPrint

## Abstract

**Background:**

A small number of prognostic and predictive tests based on gene expression are currently offered as reference laboratory tests. In contrast to such success stories, a number of flaws and errors have recently been identified in other genomic-based predictors and the success rate for developing clinically useful genomic signatures is low. These errors have led to widespread concerns about the protocols for conducting and reporting of computational research. As a result, a need has emerged for a template for reproducible development of genomic signatures that incorporates full transparency, data sharing and statistical robustness.

**Results:**

Here we present the first fully reproducible analysis of the data used to train and test MammaPrint, an FDA-cleared prognostic test for breast cancer based on a 70-gene expression signature. We provide all the software and documentation necessary for researchers to build and evaluate genomic classifiers based on these data. As an example of the utility of this reproducible research resource, we develop a simple prognostic classifier that uses only 16 genes from the MammaPrint signature and is equally accurate in predicting 5-year disease free survival.

**Conclusions:**

Our study provides a prototypic example for reproducible development of computational algorithms for learning prognostic biomarkers in the era of personalized medicine.

## Background

Currently, a number of molecular-based prognostic and predictive tests for breast cancer are offered as laboratory services for clinical use [1,2]. Such assays, which include MammaPrint [3], OncotypeDx [4], PAM50 Breast Cancer Intrinsic Subtype Classifier [5], MapQuant Dx [6] and Theros Breast Cancer Index [7], are implemented by providing multiple gene expression measurements obtained from tissue samples to multivariate classification algorithms. Currently, published evidence on clinical validity and utility for such assays as they are offered to the patients is only available for MammaPrint and OncotypeDx; for the remainder of these tests the evidence derives from analyses performed in academic settings [2].

According to a recent report [8] from the Institute of Medicine (IOM), OncotypeDx was the most widely used among these breast cancer assays, with more than 175,000 patients tested as of mid 2011, followed by MammaPrint, used for 14,000 patients. OncotypeDX combines the expression levels of 21 genes and was developed to predict the risk of distant recurrence at 10 years for women with lymph node negative, estrogen receptor (ER) positive breast cancer [4]. MammaPrint utilizes 70 genes to report a good or bad prognosis for each patient, and was developed from microarray experiments to predict 5-year metastatic recurrence of breast cancer as a first event among ER positive and negative patients [9,10]. The MammaPrint algorithm is based on correlating the 70-gene expression profile of a patient with a stored cancer profile in order to determine a risk score for the patient.

A relative small fraction of published cancer prognostic markers have subsequently been introduced in clinical practice, despite the large number of available studies focusing on biomarkers development. A major hurdle hindering the translation of this research into clinically useful assays has been identified in the lack of rigorous criteria to report and publish tumor prognostic marker studies [8]. This issue has been addressed by introducing the REMARK guidelines, a set of recommendations for tumor marker prognostic studies, which provides the necessary framework for reporting all relevant information about prognostic marker development (i.e. study design, specimen and patient characteristics, analytical and statistical methods) [11]. Another key issue in the development of cancer biomarkers is the need for detailed and complete disclosure of all data and software [8,12,13]. This need is not specific to the development of predictive signatures from high-throughput molecular data but extends to many other branches of computational medicine and biology [14,15]. Whereas the guidelines for transparency in genomic data sharing date back a decade to the adoption of the Minimal Information About Microarray Experiments (MIAMIE) standards [16], the recent scandal leading to the decision to cancel three clinical trials based on microarray-based gene expression screening tests has dramatically underscored the need for revised genomics research criteria [17] that extend and/or integrate the REMARK and MIAME guidelines.

Maximizing the level of evidence on the spectrum of reproducibility requires complete, independent replication [18]. As measured by this criterion, neither of the two successful breast cancer assays, MammaPrint and OncotypeDX, provides a paradigmatic example of the way genomic predictors should be developed. In the case of OncotypeDX, the prediction algorithm is described in detail and can be reprogrammed, but the original datasets used for the implementation and validation [4] of the assay were never placed in the public domain. Conversely, in the case of MammaPrint, although the original discovery and validation datasets [3,19] are available, the pre-processing protocol and prediction algorithm are only partially described.

Thus the entire development, including data and code, is not available for either MammaPrint nor OncotypeDX. However, in the case of MammaPrint it is possible to undertake a transparent re-analysis of the data using an alternative approach, since the raw microarray data are available. We therefore focus here our efforts on reproducing the results of Mammaprint. We collect and organize the original MammaPrint discovery and validation data. We also coordinate the associated metadata for these experiments and develop reproducible documents for their analysis. We reproduce and implement the preprocessing described in the original manuscripts. These data represent a resource that can be used by other investigators both to verify the original claims about the MammaPrint signature and to build alternative predictors. As an example of the utility of these data, we use the MammaPrint discovery and validation data to develop an alternative signature and prognostic test for breast cancer, which is based on several two-gene comparisons [20,21]. This provides a detailed, transparent and fully reproducible example of constructing a multi-gene classifier.

## Methods

### Data assembly and code

We collected the data from the original experiments used to identify [9] and develop [10] the MammaPrint 70-gene prognostic signature as provided as additional files with the original manuscripts. We also collected from ArrayExpress [22] the dataset used to retrain this signature on the custom array currently used in the MammaPrint assay [3] as well as the independent validation cohort using the same array [19]. All of these datasets have been organized in an open resource that can be used to develop and compare prognostic signatures for breast cancer (available at http://luigimarchionni.org/breastTSP.html) and Bioconductor [23]. This resource also encompasses the R [24] code and libraries used to retrieve, pre-process, manipulate, annotate, and analyze these data. The code, fully annotated and executable, is provided in the Additional files 1 and 2. All the analyses performed in our study were based on de-identified publically available data, and they were performed in compliance to the Helsinki declaration. The research did not involve any experiment on human subjects or animals and for this reason no ethical approval was necessary.

### An example of reproducible signature development

**Figure 1:**
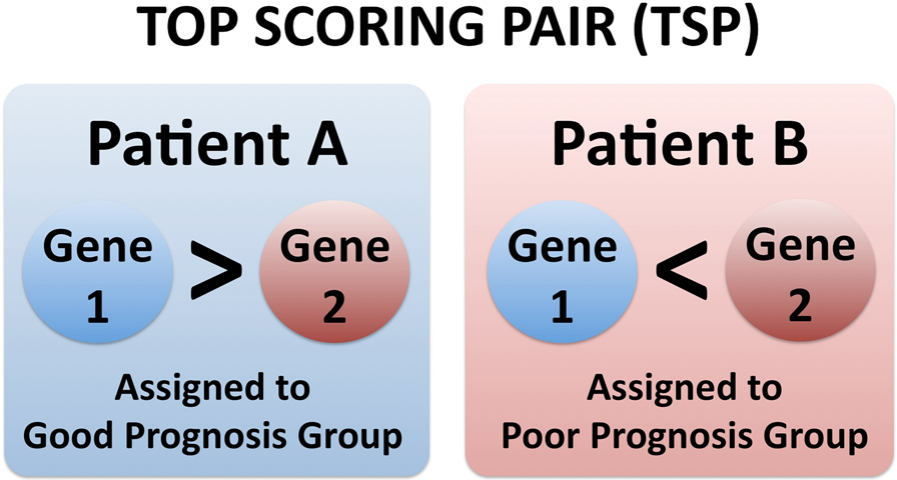
**Top Scoring Pair.** A Top Scoring Pair (TSP) is formed by a pair of measurements that consistently change ranking between samples from different prognostic groups.

In order to build our new classifier we selected the 78 patients originally used in the 70-gene prognostic signature discovery and limited our analysis to the 70 genes contained in the original signature. We made these decisions for two reasons: (a) to make our development process entirely analogous to the process for MammaPrint and (b) so that our signature can be calculated on the basis of the data from any current MammaPrint assay. To this end it should also be noted that the MammaPrint microarray platform only includes the prognostic signature genes and a set of housekeeping genes used for normalization purposes. These latter genes are designed not to change across samples and were therefore not used to train our predictor. We adopted an extension of a rank-based approach to classification called “top-scoring pairs” (TSP) for developing understandable and powerful genomic signatures. This approach is invariant to all data preprocessing and normalization steps that maintain the ordering within sample gene expression profiles. The TSP algorithm selects the pair of genes whose expression levels switch their ranking most consistently between the two prognostic groups (Figure 1). The original TSP algorithm [20] and extensions [25] have previously been successfully applied to differentiate [26], predict treatment response in breast cancer [27] and acute myeloid leukemia [28], and grade prostate cancers [29].

### Building the K-TSP classifier

We recorded the relative ordering of each pair of genes in the 70-gene MammaPrint signature in each of the 78 training samples. In other words, for each pair of genes g and g’, and for each sample j, we record whether the expression of g in sample j is larger than the expression of g’ in sample j or vice-versa. The “signature” for the TSP classifier is the pair of genes that most consistently changes its relative expression ordering between the two groups of patients and the corresponding decision rule for a new profile is determined entirely by the ordering between these two genes: choose group one if the observed ordering was most often seen in group one and group two otherwise. Here, the two groups of patients are those that recurred within 5 years (poor prognosis) and those that who did not recur (good prognosis). The K-TSP algorithm uses K pairs of genes. It proceeds by first identifying the TSP, removing these two genes from the 70-gene signature, then searching for the pair of genes among the 68 remaining that most often switch their ordering between groups, removing these from the list, and so forth. Individually, each pair of genes “votes” for one of the two groups based on the observed ordering. For a fixed number K of pairs, the final prognostic score is the sum of the votes for the poor prognosis group among all K pairs. The higher the score, the more evidence there is for poor prognosis.

### Selecting the number of pairs

**Figure 2:**
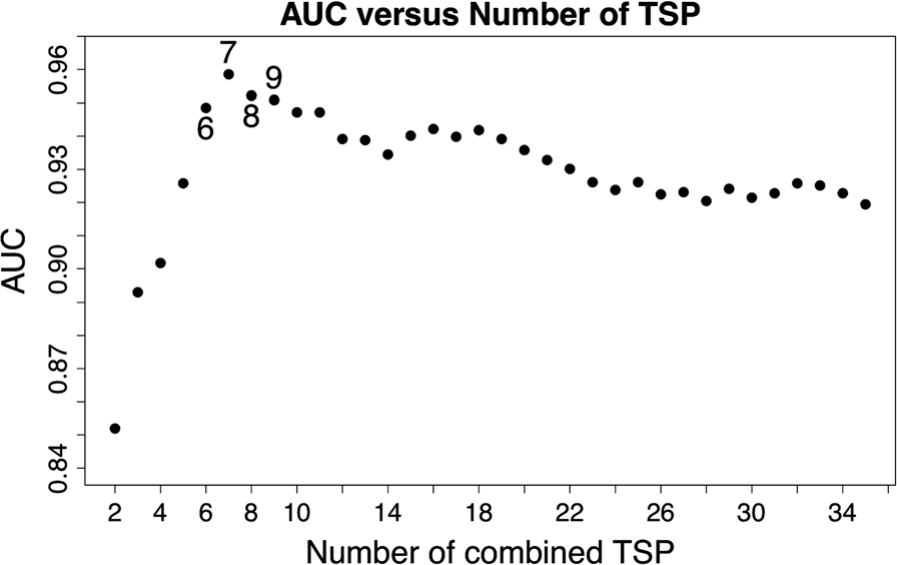
**Resubstittution performance in the training set.** Receiver Operator Characteristics (ROC) analysis was performed in the training set and the Area Under the Curve (AUC) was used to select the final number of TSPs. An 8-TSP classifier was chosen to maintain 100% training set sensitivity and maximize specificity.

**Figure 3:**
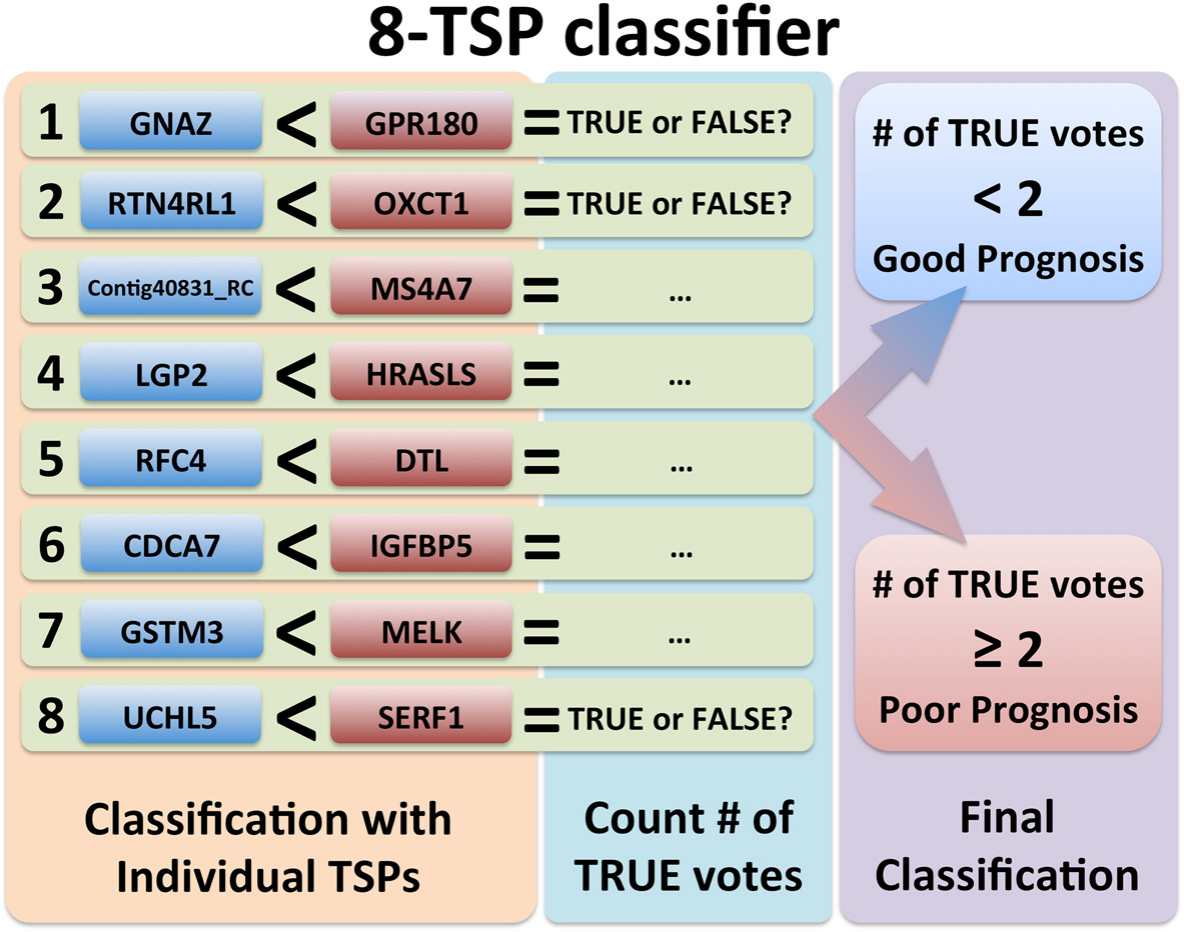
**8-TSP breast cancer prognosis signature.** Each of the 8 gene pairs votes independently; patients with two or more votes are classified as poor prognosis.

For each possible number of pairs K we measured the accuracy of the prognostic score on the training set by calculating the area under the receiver operating characteristic curve (AUC) [30] determined by considering all possible score thresholds for declaring poor prognosis. Here we used re-substitution AUC for training, since the TSP approach is based on binary decisions and is not prone to overfitting. The AUC increased with K until reaching a peak and then declined as further pairs were added (Figure 2). We focused on values of K near the peak AUC, namely K = 6 to K = 10, and only considered score thresholds achieving 100% sensitivity. The number of gene pairs K was then chosen to maximize specificity, which is equivalent to choosing the maximum score threshold which achieves 100% sensitivity. This resulted in the 8-TSP classifier (Figure 3) with score threshold two. Such resubstitution estimates obtained from the training set of samples were used only for the model optimization and do not reflect its performance, which in turn was assessed on an independent cohort of patients (see below).

### Validation of the 8-TSP signature in an independent patients cohort

To evaluate the classifier on a new sample, the relative ordering of each of the K = 8 pairs of genes is determined and the sample is assigned to the poor prognosis group if there are two or more votes for poor prognosis (Figure 3), using the same procedures previously defined in the training set of patients. The 8-TSP signature and the MammaPrint test were hence compared in terms of classification performance, using standard measures such as accuracy, sensitivity, specificity, and AUC, and in term of survival, by Kaplan-Meier and Cox regression analyses.

## Results and discussion

**Figure 4:**
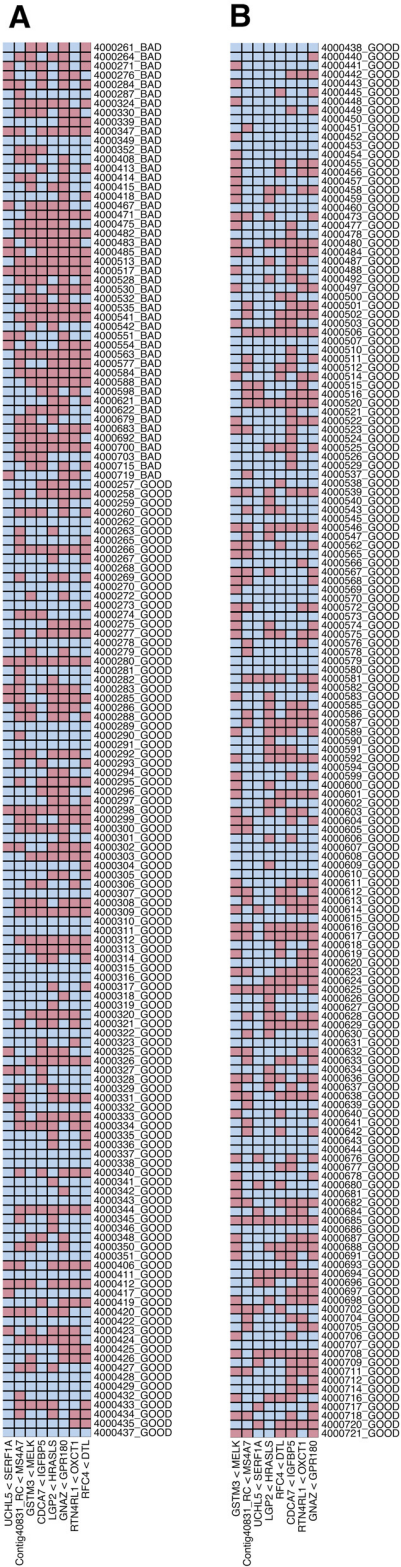
**8-TSP classification results in the validation set. Panel A)** The 8-TSP results from the first 150 patients in the validation set. Each column represents one of the 8 pairs (blue = good prognosis vote, red = bad prognosis vote) and each row is a patient. Patients with bad prognosis (top rows) have more votes for bad prognosis. **Panel B)** The 8-TSP results from the last 157 patients in the validation set.

We compared our prognostic test to the MammaPrint test based on a large independent validation cohort consisting of 307 patients from a European multi-center study [19]. In this independent validation cohort our test achieved 91% sensitivity, 47% specificity, and 69% overall accuracy (Figures 4A and 4B, and Additional files 1 and 2). Sensitivity refers to correctly classifying poor prognosis patients and specificity refers to correctly classifying good prognosis patients. For comparison, the MammaPrint prognostic test achieves 89% sensitivity, 42% specificity, and 65% overall accuracy [19,31] in this same validation set. Such performance in predicting metastatic recurrence within 5 years was reflected in the AUC estimates: 0.69 (95% CI: 0.64 − 0.74) and 0.59 (95% CI: 0.55 − 0.62) for the 8-TSP and MammaPrint respectively. (Comparable results were obtained by PAM, a well-known classification method; see the Additional files 1 and 2.) Finally, while in the prediction of a metastatic event within five years the 8-TSP classifier performed better than the MammaPrint test, this latter assay maintained a better performance at later time points as revealed in survival analyses. This finding probably indicates that the additional features of the 70-gene signature not used in the 8-TSP classifier might carry additional prognostic information beyond five years (see Additional files 1 and 2).

We have therefore built a prognostic classifier based on the genes from the MammaPrint signature that is as accurate in predicting 5-year disease-free survival as the MammaPrint prognostic test based. Our classifier only requires the measurement of expression for 16 of the 70 genes used in Mammaprint. Moreover, the new test is easy to interpret and is robust with respect to any preprocessing of the expression data that maintains the ordering among expression levels within sample profiles.

Finally, all design decisions and choices of parameters were based entirely on the training set. There was no “data leakage”: no test data was examined until all aspects of classifier development were “locked up.” These are considered critical steps in developing reproducible and accurate genomic signatures as defined by the IOM report [8]. The two key parameters are K, the number of pairs of genes in the signature, and the score threshold. We only considered values of K between 6 and 10 since these values maximized overall performance, and we only considered thresholds that obtained 100% sensitivity. Under these design constraints, we selected the K = 8 since this value maximized specificity at 100% sensitivity (Figure 2). Our final classifier labels a sample as poor prognosis if two or more among the 8 pairs votes for the poor prognosis group (Figure 3).

Our 8-TSP signature can be viewed as the combination of multiple coordinated biological processes. Of the 70 genes originally identified in the study by van’t Veer and colleagues [10], 18 genes had expression values positively associated with good prognosis, while 52 were associated with metastatic recurrence. Four of the K = 8 pairs combine genes positively correlated with good prognosis (RTN4RL1, LGP2, MS4A7, and GSTM3) with genes associated with bad prognosis (OXCT1, HRASLS, Contig40831_RC, and MELK). These pairs represent a coordinated change from good prognosis expression patterns to poor prognosis patterns across multiple gene pairs. The remaining pairs comprise only genes originally associated with a poor prognosis (GPR180, DTL, IGFBP5, SERF1A, GNAZ, RFC4, CDCA7, and UCHL5), suggesting that it is the quantitative level of expression of these genes that is important for predicting prognosis.

It is of note that each individual TSP involved in the final classification scheme can be viewed as a separate molecular switch between the two prognostic groups, possibly entailing also a mechanistic underpinning. To this end some of the pairs we have identified appear to have an additional underlying mechanistic biological relationship. For instance one of the gene pairs, DTL-RCF4, appears to be tightly associated with the regulation of the replication fork and the DNA damage response. DTL and RCF4 physically interact and modulate the activity of the proliferating cell nuclear antigen (PCNA) [32-34], which plays a central role in the coordination of these processes. Similarly, another pair, GPR180-GNAZ, code for proteins involved in G protein mediated cellular signaling.

## Conclusions

Our goal was to provide a transparent example of the manner in which a genomics-based cancer predictor might be developed from training data and evaluated on independent test data with sufficient detail and documentation to allow the full process to be replicated by other researchers. Due to the unavailability of the original data, it was not possible carry out this process for OncotypeDX, which is presently the most used and validated predictor of this kind. Consequently, we performed a re-analysis of MammaPrint data. To this end, we selected the same samples and end-point originally used for the implementation of this assay, although we are aware that a stratified analysis across ER positive and negative patients would be much more appropriate. In order to illustrate the development process from end to end, including a transparent decision rule, we have introduced a more parsimonious classifier with sensitivity, specificity, and overall accuracy very similar to the 70-gene MammaPrint signature.

Our analysis was performed in complete adherence to the principles of transparent and reproducible research [13,18], providing all data sources used, and the complete code and software necessary for data preprocessing, analysis and validation. To our knowledge, this is one of the few, if not the first, development of a genomic signature adhering to these standards.

## Abbreviations

TSP: Top scoring pair; ROC: Receiver operator curve; AUC: Area under the curve; FDA: Food and drug administration; IOM: Institute of medicine; ER: Estrogen receptor; REMARK: Reporting recommendations for tumour marker prognostic studies; MIAMIE: Minimal information about microarray experiments; CI: Confidence intervals; RTN4RL1: Reticulon 4 receptor-like 1; LGP2: DHX58 DEXH (ASP-GLU-X-HIS) box polypeptide 58; MS4A7: MS4A7 membrane-spanning 4-domains, subfamily A, member 7; GSTM3: Glutathione S-Transferase MU 3 (BRAIN); OXCT1: 3-oxoacid coa transferase 1; HRASLS: HRAS-Like suppressor; MELK: Maternal embryonic leucine zipper kinase; GPR180: G Protein-coupled receptor 180; DTL: Denticleless E3 ubiquitin protein ligase homolog (drosophila); IGFBP5: Insulin-like growth factor binding protein 5; SERF1A: Small edrk-rich factor 1A (TELOMERIC); GNAZ: Guanine nucleotide binding protein (G protein), alpha Z polypeptide; RFC4: Replication factor C (activator 1) 4, 37KDA; CDCA7: Cell division cycle associated 7; UCHL5: Ubiquitin carboxyl-terminal hydrolase L5; PCNA: Proliferating cell nuclear antigen.

## Competing interests

The authors declare that they have no competing interests.

## Authors’ contributions

LM, JTL, and DG conceived the study; LM performed all the analysis and assembled all the datasets; LM and BA implemented the software packages used in the analysis; LM, JTL and DG wrote the manuscript. All authors read and approved the manuscript.

## Supplementary Material

Additional file 1 Fully reproducible vignette of the analysis. Click here for file

Additional file 2 **The archive contains the following files: “bmc_article.bst”: BMC series bibliography style; “localFiles/contactAgendia": instructions to obtain the hybridization mapping information from Agendia; “objs/buyseEset.rda”: ExpressionSet for the Buyse cohort; “objs/glasEset.rda”: ExpressionSet for the Glas cohort; “Supplement.bib”: Bibliography in BibTex format; “Supplement.Rnw”: Rnoweb/Sweave file containing code and text used to create the “Supplement.tex” file; “Supplement.tex”: LaTeX file resulting from running the Sweave with the “Supplement.Rnw” file; All source code, data, and software packages used in the analyses are also available for download online from: **http://luigimarchionni.org/breastTSP.html. Click here for file

## References

[B1] Marchionni L Wilson RF Wolff AC Marinopoulos S Parmigiani G Bass EB Goodman SN Systematic review: gene expression profiling assays in early-stage breast cancer Ann Intern Med 2008 148 5 358 369 10.7326/0003-4819-148-5-200803040-00208 18252678

[B2] Paik S Is gene array testing to be considered routine now? Breast 2011 20 Suppl 3 S87 S91 22015300 10.1016/S0960-9776(11)70301-0

[B3] Glas AM Floore A Delahaye LJ Witteveen AT Pover RC Bakx N Lahti-Domenici JS Bruinsma TJ Warmoes MO Bernards R Converting a breast cancer microarray signature into a high-throughput diagnostic test BMC Genomics 2006 7 278 10.1186/1471-2164-7-278 17074082 PMC1636049

[B4] Paik S Shak S Tang G Kim C Baker J Cronin M Baehner FL Walker MG Watson D Park T A multigene assay to predict recurrence of tamoxifen-treated, node-negative breast cancer N Engl J Med 2004 351 27 2817 2826 10.1056/NEJMoa041588 15591335

[B5] Parker JS Mullins M Cheang MC Leung S Voduc D Vickery T Davies S Fauron C He X Hu Z Supervised risk predictor of breast cancer based on intrinsic subtypes J Clin Oncol 2009 27 8 1160 1167 10.1200/JCO.2008.18.1370 19204204 PMC2667820

[B6] Loi S Haibe-Kains B Desmedt C Lallemand F Tutt AM Gillet C Ellis P Harris A Bergh J Foekens JA Definition of clinically distinct molecular subtypes in estrogen receptor-positive breast carcinomas through genomic grade J Clin Oncol 2007 25 10 1239 1246 10.1200/JCO.2006.07.1522 17401012

[B7] Ma XJ Salunga R Dahiya S Wang W Carney E Durbecq V Harris A Goss P Sotiriou C Erlander M A five-gene molecular grade index and HOXB13:IL17BR are complementary prognostic factors in early stage breast cancer Clin Cancer Res 2008 14 9 2601 2608 10.1158/1078-0432.CCR-07-5026 18451222

[B8] IOM (Institute of Medicine) Evolution of translational Omics: lessons learned and the path forward 2012 Washington, D.C: The National Academy Press 24872966

[B9] van’t Veer LJ Dai H van de Vijver MJ He YD Hart AA Mao M Peterse HL van der Kooy K Marton MJ Witteveen AT Gene expression profiling predicts clinical outcome of breast cancer Nature 2002 415 6871 530 536 10.1038/415530a 11823860

[B10] van de Vijver MJ He YD van’t Veer LJ Dai H Hart AA Voskuil DW Schreiber GJ Peterse JL Roberts C Marton MJ A gene-expression signature as a predictor of survival in breast cancer N Engl J Med 2002 347 25 1999 2009 10.1056/NEJMoa021967 12490681

[B11] McShane LM Altman DG Sauerbrei W Taube SE Gion M Clark GM Reporting recommendations for tumor marker prognostic studies (REMARK) J Natl Cancer Inst 2005 97 16 1180 1184 10.1093/jnci/dji237 16106022

[B12] Leek JT Peng RD Anderson RR Personalized medicine: keep a way open for tailored treatments Nature 2012 484 7394 318 22517151 10.1038/484318a PMC3375895

[B13] Baggerly K Disclose all data in publications Nature 2010 467 7314 401 20864982 10.1038/467401b

[B14] Peng RD Reproducible research and biostatistics Biostatistics 2009 10 3 405 408 10.1093/biostatistics/kxp014 19535325

[B15] Peng RD Dominici F Zeger SL Reproducible epidemiologic research Am J Epidemiol 2006 163 9 783 789 10.1093/aje/kwj093 16510544

[B16] Brazma A Hingamp P Quackenbush J Sherlock G Spellman P Stoeckert C Aach J Ansorge W Ball CA Causton HC Minimum information about a microarray experiment (MIAME)-toward standards for microarray data Nat Genet 2001 29 4 365 371 10.1038/ng1201-365 11726920

[B17] Goozner M Duke scandal highlights need for genomics research criteria J Natl Cancer Inst 2011 103 12 916 917 10.1093/jnci/djr231 21693754

[B18] Peng RD Reproducible research in computational science Science 2012 334 6060 1226 1227 22144613 10.1126/science.1213847 PMC3383002

[B19] Buyse M Loi S van’t Veer L Viale G Delorenzi M Glas AM d'Assignies MS Bergh J Lidereau R Ellis P Validation and clinical utility of a 70-gene prognostic signature for women with node-negative breast cancer J Natl Cancer Inst 2006 98 17 1183 1192 10.1093/jnci/djj329 16954471

[B20] Geman D d'Avignon C Naiman DQ Winslow RL Classifying gene expression profiles from pairwise mRNA comparisons Stat Appl Genet Mol Biol 2004 3 Article 19 10.2202/1544-6115.1071 PMC1989150 16646797

[B21] Leek JT The tspair package for finding top scoring pair classifiers in R Bioinformatics 2009 25 9 1203 1204 10.1093/bioinformatics/btp126 19276151 PMC2672632

[B22] Brazma A Kapushesky M Parkinson H Sarkans U Shojatalab M Data storage and analysis in ArrayExpress Methods Enzymol 2006 411 370 386 16939801 10.1016/S0076-6879(06)11020-4

[B23] A simple and reproducible breast cancer prognostic test http://luigimarchionni.org/breastTSP.html 10.1186/1471-2164-14-336 PMC3662649 23682826

[B24] Ihaka R Gentleman R R: A language for data analysis and graphics J Comput Graph Stat 1996 5 299 314

[B25] Tan AC Naiman DQ Xu L Winslow RL Geman D Simple decision rules for classifying human cancers from gene expression profiles Bioinformatics 2005 21 20 3896 3904 10.1093/bioinformatics/bti631 16105897 PMC1987374

[B26] Price ND Trent J El-Naggar AK Cogdell D Taylor E Hunt KK Pollock RE Hood L Shmulevich I Zhang W Highly accurate two-gene classifier for differentiating gastrointestinal stromal tumors and leiomyosarcomas Proc Natl Acad Sci U S A 2007 104 9 3414 3419 10.1073/pnas.0611373104 17360660 PMC1805517

[B27] Weichselbaum RR Ishwaran H Yoon T Nuyten DS Baker SW Khodarev N Su AW Shaikh AY Roach P Kreike B An interferon-related gene signature for DNA damage resistance is a predictive marker for chemotherapy and radiation for breast cancer Proc Natl Acad Sci U S A 2008 105 47 18490 18495 10.1073/pnas.0809242105 19001271 PMC2587578

[B28] Raponi M Lancet JE Fan H Dossey L Lee G Gojo I Feldman EJ Gotlib J Morris LE Greenberg PL A 2-gene classifier for predicting response to the farnesyltransferase inhibitor tipifarnib in acute myeloid leukemia Blood 2008 111 5 2589 2596 10.1182/blood-2007-09-112730 18160667

[B29] Carro MS Lim WK Alvarez MJ Bollo RJ Zhao X Snyder EY Sulman EP Anne SL Doetsch F Colman H The transcriptional network for mesenchymal transformation of brain tumours Nature 2010 463 7279 318 325 10.1038/nature08712 20032975 PMC4011561

[B30] van Belle G Fisher LD Heagerty PJ Lumley T Biostatistics: A methodology for the health sciences 2004 2 Hoboken, New Jersey: John Wiley and Sons

[B31] Tian S Roepman P Van't Veer LJ Bernards R de Snoo F Glas AM Biological functions of the genes in the mammaprint breast cancer profile reflect the hallmarks of cancer Biomark Insights 2010 5 129 138 21151591 10.4137/BMI.S6184 PMC2999994

[B32] Zhang G Gibbs E Kelman Z O'Donnell M Hurwitz J Studies on the interactions between human replication factor C and human proliferating cell nuclear antigen Proc Natl Acad Sci U S A 1999 96 5 1869 1874 10.1073/pnas.96.5.1869 10051561 PMC26703

[B33] Ohta S Shiomi Y Sugimoto K Obuse C Tsurimoto T A proteomics approach to identify proliferating cell nuclear antigen (PCNA)-binding proteins in human cell lysates. Identification of the human CHL12/RFCs2-5 complex as a novel PCNA-binding protein J Biol Chem 2002 277 43 40362 40367 10.1074/jbc.M206194200 12171929

[B34] Jascur T Fotedar R Greene S Hotchkiss E Boland CR N-methyl-N'-nitro-N-nitrosoguanidine (MNNG) triggers MSH2 and Cdt2 protein-dependent degradation of the cell cycle and mismatch repair (MMR) inhibitor protein p21Waf1/Cip1 J Biol Chem 2011 286 34 29531 29539 10.1074/jbc.M111.221341 21725088 PMC3190993

