# Supplementary material for: A simple and reproducible breast cancer prognostic test
Source: BMC Genomics. 2013 May 17;14:336. doi: 10.1186/1471-2164-14-336 (PMC3662649; doi:10.1186/1471-2164-14-336)
Supplement: Additional file 1 — Fully reproducible vignette of the analysis. [file 1471-2164-14-336-S1.pdf]

Supplemental Data

"A simple and reproducible breast cancer prognostic test"

Luigi Marchionni[1], Bahman Afsari[4], Donald Geman[3,4], and Jeffrey T. Leek[2]

[1]The Sidney Kimmel Comprehensive Cancer Center, Johns Hopkins University School of Medicine
[2]Department of Biostatistics, Johns Hopkins Bloomberg School of Public Health
[3]Institute for Computational Medicine, Johns Hopkins University
[4]Department of Applied Mathematics and Statistics, Johns Hopkins University

April 8, 2013

Contents

1 Overview **4**

2 `R-Bioconductor` analytical packages **4**

3 Gene expression and clinical data preparation **6**
3.1 The `mammaPrintData` `R-Bioconductor` package . . . 6
3.2 Glas cohort: ArrayExpress "E-TABM-115" series . . . 7
3.2.1 Dye-swap hybridization pairs identification . . . 7
3.2.2 Gene expression correction and summarization . . . 10
3.3 Buyse cohort: ArrayExpress "E-TABM-77" series . . . 14
3.3.1 Gene expression correction and summarization . . . 14
3.4 End-point and prognostic groups selection . . . 17
3.4.1 Training set: Glas cohort (E-TABM-115) . . . 17
3.4.2 Test set: Buyse cohort (E-TABM-77) . . . 19
3.5 Comparing the clinical information across studies and cohorts . . . 20
3.5.1 The van't Veer cases in the Glas study . . . 21
3.5.2 The Van de Vijver cases in the Glas study . . . 22

4 Prognostic predictor training **23**
4.1 K-TSP classifier development using the Glas data set . . . 24
4.1.1 Definition of the k-TSP classifier training functions . . . 24
4.1.2 k optimization based on resubstitution AUC . . . 25
4.2 Re-training of the MammaPrint classifier . . . 31
4.3 Development of other classifiers using the Glas data set . . . 32
4.3.1 LDA classifier training . . . 32
4.3.2 PAM classifier training . . . 33
4.4 ROC-AUC analysis in the Glas cohort . . . 35

5 Prognostic predictor validation **36**
5.1 k-TSP classifier performance in the Buyse cohort . . . 36

5.2 MammaPrint assay performance in the Buyse cohort . . . 39
5.2.1 Reported MammaPrint predictions . . . 39
5.2.2 Retrained MammaPrint predictions . . . 40
5.3 Performance of other classifiers in the Buyse cohort . . . 41
5.3.1 LDA performance in the Buyse cohort . . . 41
5.3.2 PAM performance in the Buyse cohort . . . 41
5.4 Performance of clinical classifiers in the Buyse cohort . . . 42
5.4.1 Adjuvan! online performance in the Buyse cohort . . . 42
5.4.2 NPI performance in the Buyse cohort . . . 43
5.4.3 St Gallen performance in the Buyse cohort . . . 43
5.5 ROC-AUC analysis in the Buyse cohort . . . 44
5.6 Survival analysis in the Buyse cohorts . . . 45
5.6.1 Time to development of metastasis . . . 45
5.6.2 Disease free survival . . . 50
5.6.3 Overall survival . . . 55
5.6.4 Summary of Cox proportional hazards models analysis . . . 60
5.7 Stratification by estrogen receptor status . . . 60

6 System information **62**

7 References **63**

List of Tables

1 The mammaPrintData package content . . . 7
2 The seventyGeneData package content . . . 21
3 Cases from the Glas study missing or with different clinical information in the van't Veer study 22
4 Cases from the van't Veer study missing or with different clinical information in the Glas study 22
5 ROC parameters for the *k*-TSP classifier combining the top 5 TSPs . . . 26
6 ROC parameters for the *k*-TSP classifier combining the top 6 TSPs . . . 27
7 ROC parameters for the *k*-TSP classifier combining the top 7 TSPs . . . 27
8 ROC parameters for the *k*-TSP classifier combining the top 8 TSPs . . . 27
9 ROC parameters for the *k*-TSP classifier combining the top 9 TSPs . . . 27
10 Individual TSP contained in the final classifier. . . . 28
11 Cox proportional hazard model results . . . 60

List of Figures

1 AUC for increasing *k*-TSP combinations . . . 26
2 ROC curves for the best *k*-TSP combinations . . . 28
3 Individual TSP predictions: Glas dataset . . . 30
4 PAM shrunken centroids . . . 33
5 PAM misclassification error . . . 34
6 ROC-AUC analysis in the training set: Glas cohort . . . 35
7 Individual TSP predictions: Buyse dataset (first set) . . . 37
8 Individual TSP predictions: Buyse dataset (second set) . . . 38
9 ROC-AUC analysis in the test set: Buyse cohort . . . 45

10 Kaplan-Meier curves for k-TSP prediction: time to development of metastasis. 47
11 Kaplan-Meier curves for the mammaPrint prediction: time to development of metastasis. . . . 48
12 Kaplan-Meier curves for the LDA prediction: time to development of metastasis. 49
13 Kaplan-Meier curves for the PAM prediction: time to development of metastasis. 50
14 Kaplan-Meier curves for k-TSP prediction: disease free survival. 52
15 Kaplan-Meier curves for mammaPrint prediction: disease free survival. 53
16 Kaplan-Meier curves for LDAprediction: disease free survival. 54
17 Kaplan-Meier curves for PAMprediction: disease free survival. 55
18 Kaplan-Meier curves for k-TSP prediction: overall survival. 56
19 Kaplan-Meier curves for mammaPrint prediction: overall survival. 57
20 Kaplan-Meier curves for LDA prediction: overall survival. 58
21 Kaplan-Meier curves for PAM prediction: overall survival. 60

1 Overview

In the present study we analyzed the expression of the genes constituting the 70-gene breast cancer prognostic signature [1, 2], as implemented in the MammaPrint assay [3, 4], to develop novel and simple prognostic predictors based on the Top-Scoring-Pair (TSP) algorithm [5, 6]. In compliance to recently suggested policies for reproducible research in computational science [7], this document contains the complete `R` code used to:

1. Download and install the data sets used in the manuscript:
   (a) the Glas data set (ArrayExpress [8] "E-TABM-115"), analyzed on the 1.9k MammaPrint array combining the two original cohorts above;
   (b) the Buyse data set (ArrayExpress "E-TABM-77"), a multicenter validation cohort analyzed on the 1.9k MammaPrint array;
   (c) the original van't Veer data set, analyzed on the two-colors Agilent/Rosetta 24k array;
   (d) the orignal Vand de Vijver data set, analyzed on the two-colors Agilent/Rosetta 24k array;
2. Pre-process and normalize all gene expression data as necessary;
3. Obtain the 70-gene list used to implement the MammaPrint assay;
4. Analyze the data using the Top-Scoring-Pair (TSP) and the k-TSP algorithms [5, 6].

For a compete review of these breast cancer studies see Marchionni et al [9, 10]. The complete `R` code, the libraries used to perform all analyses, and the Sweave file used to produce this document are available from the website accompanying this manuscript (`http://luigimarchionni.org/breastTSP.html`).

2 `R-Bioconductor` analytical packages

The following `R` packages were used to perform our analyses and produce this vignette:

- `switchBox`: a library that implements the methods and classes necessary to perform the k-TSP classification [5, 6]. This package can be currently obtained from the website accompanying this manuscript (`http://luigimarchionni.org/breastTSP.html`), and will be also available with the next `Bioconductor` release;
- `survival`: this package implements classes and methods to perform survival analysis, and it is part of the base `R` installation;
- `impute`: this library implements several methods to impute missing values in gene expression matrices [11], and can be obtained from `Bioconductor`;
- `MLInterfaces`: this package provides a uniform interface to the many classification functions available in `R` and can be obtained from `Bioconductor`;
- `pamr`: this library provides an `R` implementation of the Prediction Analysis for Microarray method described by Tibshirani et al [12] and can be obtained from `Bioconductor`;
- `org.Hs.eg.db`: this library contains *Homo sapiens* gene annotation and can be obtained from `Bioconductor`;

- pROC: this package implements classes methods to perform Receiver Operator Curve (ROC) analysis [13], and can be obtained from CRAN;
- caret: this library implements miscellaneous functions for training and plotting classification and regression models, and can be obtained from CRAN;
- xtable: this package allows to output nicely formatted tables in LaTeXand can be obtained from CRAN;
- e1071: this package contains miscellaneous statistial and graphical functions required by the caret package and can be obtained from CRAN;
- gplots: this library contains the read.xls function enabling to directly read excel spreadsheet into an Rsession and can be obtained from CRAN;
- lsa: this package contains cosine function to compute the cosine similarity between two vectors, and can be obtained from CRAN;

The chunks of R code below can be used to obtain and install all necessary packages from CRAN, Bioconductor, or the website repository accompanying this manuscript.

Installing from Bioconductor:

```
> ###Source the biocLite.R script from Bioconductor
> source("http://bioconductor.org/biocLite.R")
> ###Get the list of available packages
> installedPckgs <- installed.packages()[,"Package"]
> ###Define the list of desired libraries
> pckgListBIOC <- c("impute", "org.Hs.eg.db", "MLInterfaces", "pamr")
> ###Load the packages, install them from Bioconductor if needed
> for (pckg in pckgListBIOC) {
        if (! pckg %in% installedPckgs) biocLite(pckg)
        require(pckg, character.only=TRUE)
 }
```

Installing from CRAN:

```
> ###Define the repository
> nciRepos <- "http://watson.nci.nih.gov/cran_mirror"
> ###Define the list of desired libraries
> pckgListCRAN <- c("pROC", "caret", "xtable", "gplots", "e1071", "lsa")
> ###Load the packages, install them from CRAN if needed
> for (pckg in pckgListCRAN) {
        if (! pckg %in% installedPckgs) install.packages(pckg, repos=nciRepos)
        require(pckg, character.only=TRUE)
 }
```

Installing from the manuscript website:

```
> ###Define the repository
> marchionniRepos <- "http://luigimarchionni.org/software/"
> ###Define the list of desired libraries
> pckgListMS <- c("switchBox")
> ###Load the packages, install them from Marchionni's repository
> for (pckg in pckgListMS) {
        if (! pckg %in% installedPckgs) install.packages(pckg, repos=marchionniRepos)
        require(pckg, character.only=TRUE)
 }
```

3 Gene expression and clinical data preparation

3.1 The mammaPrintData R-Bioconductor package

The original data sets used to redevelop [3] the 70-gene prognostic signature into the MammaPrint assays, and then to validate it [4], can be obtained from the ArrayExpress database [8]:

- http://www.ebi.ac.uk/arrayexpress/experiments/E-TABM-77
- http://www.ebi.ac.uk/arrayexpress/experiments/E-TABM-115

These two gene expression series account for clinical information, microarray features annotation, and complete unprocessed raw gene expression data. The pre-processed, normalized, and summarized values used by the authors in their studies were not included in the ArrayExpress submission, hence data pre-processsing is required prior any analysis can be performed. For our study we have therefore retrieved the E-TABM-115 and E-TABM-77 series from ArrayExpress, and we have assembled the data into the mammaPrintData R-Bioconductor package. This R library accounts for expression values, clinical information, and microarray features annotation for the 1.9k MammaPrint microarray, including the mapping information for the 231-gene originally associated with patient's outcome, and for the 70-gene breast cancer progostic signature (see the mammaPrintData package vignette for details). Since a two-colors dye-swap design was used in these two studies [3, 4], our experiment data R-Bioconductor package contains four RGList-Class instances, two for each cohort, and two for each dye-swap replicate, as follows:

- glasRGcy5: the RGList-Class instance for the hybridizations of the Glas' cohort in which the information provided was associated with the with Cy5 channel;
- glasRGcy3: the RGList-Class instance for the hybridizations of the Glas' cohort in which the information provided was associated with the Cy3 channel;
- buyseRGcy5: the RGList-Class instance for the hybridizations of the Buyse's cohort in which the reference RNA was labeled with Cy5;
- buyseRGcy3: the RGList-Class instance for the hybridizations of the Buyse's cohort in which the reference RNA was labeled with Cy3;

The mammaPrintData package vignette provides all the links to the original data sources, as well as the complete R code used to assemble the RGList-Class objects contained in the package. This Rlibrary will be made available with the next Bioconductorrelease, and can be currently dowloaded and installed from the website accompanying this manuscript (http://luigimarchionni.org/breastTSP.html), as shown below:

```
> ###Obtain and install the data package from the manuscript website
> marchionniRepos <- "http://luigimarchionni.org/software/"
> install.packages("mammaPrintData", repos=marchionniRepos)
```

The content of the mammaPrintData R-Bioconductor package is shown in Table 1.

Table 1: The mammaPrintData package content

Package	Item	Title
mammaPrintData	buyseRGcy3 (buyseRG)	Gene expression, annotations and clinical information for the Buyse cohort: set of dye-swap hybridizations in which the reference RNA was labeled with Cy3
mammaPrintData	buyseRGcy5 (buyseRG)	Gene expression, annotations and clinical information for the Buyse cohort: set of dye-swap hybridizations in which the reference RNA was labeled with Cy5
mammaPrintData	glasRGcy3 (glasRG)	Gene expression, annotations and clinical information for the Glas cohort: set of dye-swap hybridizations in which the information was associated with RNA samples labeled with Cy3
mammaPrintData	glasRGcy5 (glasRG)	Gene expression, annotations and clinical information for the Glas cohort: set of dye-swap hybridizations in which the information was associated with RNA samples labeled with Cy5

3.2 Glas cohort: ArrayExpress "E-TABM-115" series

The data set used in the Glas study [3] combined a large proportion of cases from the original van't Veer and Van de Vijver cohorts. This data set was used for the implementation of the original 70-gene breast cancer prognostic signature into the MammaPrint assay. In particular this cohort accounts for 162 unique breast cancer cases, which were analyzed using a dye-swap two-colors design, comparing each sample against the same reference RNA (162 samples * 2 channels * 2 dye-swap hybridizations = 648 total data points).

```
> ###Load the Glas cohort data
> data(glasRG)
> ###Dimensions of the RGList objects for each set of swapped hybridizations
> dim(glasRGcy5)

[1] 1900  162

> dim(glasRGcy3)

[1] 1900  162
```

3.2.1 Dye-swap hybridization pairs identification

As described in details in the `mammaPrintData` package vignette, the phenotypic and clinical information associated with the ArrayExpress "E-TABM-115" series available on Apr 08, 2013 was incomplete. The SDRF annotation table, in fact, did not account for a total of 648 rows, one for each hybridization channel as expected (162 samples for 2 channels, for 2 replicated dye-swap hybridizations), but only for 324 rows, one for each hybridization result file. Based on this SDRF annotation table it was possible to associate the clinical information provided only for a half of the results files, corresponding to one set of the dye-swap hybridization technical replicates performed. For the second set of replicates only the reference RNA information was available. Such phenotypic information, as it is stored in the `RGList-class` instances of the `mammaPrintData` package is shown below.

```
> ###Phenotypic information provided with the E-TABM-115 serries
> colnames(glasRGcy3$targets)

 [1] "Source.Name"
 [2] "Characteristics.EventDistantMetastases"
 [3] "Characteristics.EventDeath"
 [4] "Characteristics.PeriodTillDevelopmentOfDistantMetastases.years"
 [5] "Characteristics.OverallSurvival"
```

```
 [6] "Sample.Name"
 [7] "Extract.Name"
 [8] "Labeled.Extract.Name"
 [9] "Label"
[10] "Hybridization.Name"
[11] "Factor.Value.overall_survival"
[12] "Factor.Value.Event_Death"
[13] "Factor.Value.Event_distant_metastases"
[14] "Factor.Value.Time_to_development_of_distant_metastases"
[15] "Scan.Name"
[16] "Array.Data.File"
[17] "putativeCohort"
```

The R code chunk below shows the phenotypic information associated with the first set of dye-swap hybridizations, which is stored in the `glasRGcy3$targets` object, and correspond to the clinical information for the patients analyzed in the Glas study:

```
> ###Selection of column containing clinical information: i.e. overall survival
> colSelection <- c("Source.Name", "Factor.Value.overall_survival", "Scan.Name")
> ###The phenotypic information for 4 hybridizations from the FIRST dye-swap set
> head(glasRGcy3$targets[, colSelection], n=4)
```

```
    Source.Name Factor.Value.overall_survival            Scan.Name
86          MRP                reference pool US22502555_967_2_397
4           MRP                reference pool US22502555_953_2_366
67          MRP                reference pool US22502555_952_2_344
122         MRP                reference pool US22502555_951_2_339
```

On the contrary the R code chunk below shows the phenotypic information associated with the second set of dye-swap hybridizations, which is stored in the `glasRGcy5$targets` object, and correspond to the information about the "MRP" reference RNA used in the study. For this reason the correspondence between patients' clinical information and the result files for this second set of hybridizations and is unknown.

```
> ###The phenotypic information for 4 hybridizations from the SECOND dye-swap set
> head(glasRGcy5$targets[, colSelection], n=4)
```

```
    Source.Name Factor.Value.overall_survival            Scan.Name
258     9671397                    8.77 years US22502555_967_1_397
81      9531366                   17.15 years US22502555_953_1_366
55      9521344                    6.87 years US22502555_952_1_344
121     9511339                   16.59 years US22502555_951_1_339
```

In order to analyze the complete Glas data set it was necessary ot link together each pair of hybridization result files corresponding to a dye-swap pair of technical replicates. For the present study this mapping information was kindly provided by Dr. Glas, the corresponding author of the original manuscript [3].

Processed gene expression data using such information are available from the website accompanying this manuscript (`http://luigimarchionni.org/breastTSP.html`).

```
> ###Load the gdata library to read Excel spreadsheets
> require(gdata)
> ###Read in mapping informaiton spreadsheet file obtained from Dr Glas
> localFiles <- list.files("./localFiles/")
> if ("AgendiaExeriment overview_E_TAB_115.xlsx" %in% localFiles) {
         mapInfo <- read.xls("./localFiles/AgendiaExeriment overview_E_TAB_115.xlsx",
                             header=TRUE, stringsAsFactors=FALSE)
 } else {
         mapInfo <- "Obtain mapping information from Agendia or use the provided normalized data."
         print(mapInfo)
 }
```

The chunk of R output below shows an excerpt from the mapInfo data.frame, in which each pair of hybridizations constituting a dye-swap pair is clearly identified.

```
> ###Dimensions of the mapping information data.frame
> dim(mapInfo)

[1] 324   4

> ###Show the first 4 rows of the mapping information data.frame
> head(mapInfo, n=4)

                          Hybridization     Cy5     Cy3 polarity
1 US22502555_16011886011186_S01_1_1.txt 1118611     MRP        +
2 US22502555_16011886011186_S01_1_2.txt     MRP 1118611        -
3 US22502555_16011886011186_S01_1_3.txt 1118613     MRP        +
4 US22502555_16011886011186_S01_1_4.txt     MRP 1118613        -
```

The R code chunk below was used to count the number of times the “MRP” reference RNA was hybridized in each channel (162 pairs of dys-swap hibridizations).

```
> ###Count channel information versus reference RNA
> table(ReferenceRNAinCy3 = mapInfo$Cy3 %in% "MRP", ReferenceRNAinCy5 = mapInfo$Cy5 %in% "MRP" )

                 ReferenceRNAinCy5
ReferenceRNAinCy3 FALSE TRUE
            FALSE     2  160
            TRUE    162    0

> ###Two Cy5 values in "mapInfo" are equal to empty strings rather thant "MRP"
> mapInfo$Cy5[ ! mapInfo$Cy3 %in% "MRP" & ! mapInfo$Cy5 %in% "MRP"]

[1] "" ""

> ###Assign the reference RNA "MRP" value to such empty strings
> mapInfo$Cy5[ ! mapInfo$Cy3 %in% "MRP" & ! mapInfo$Cy5 %in% "MRP"] <- "MRP"
> ###Re-count channel information versus reference RNA
> table(ReferenceRNAinCy3 = mapInfo$Cy3 %in% "MRP", ReferenceRNAinCy5 = mapInfo$Cy5 %in% "MRP" )

                 ReferenceRNAinCy5
ReferenceRNAinCy3 FALSE TRUE
            FALSE     0  162
            TRUE    162    0
```

Using this mapping information contained in the mapInfo data.frame we could eventually link the phenotypic information contained in the glasRGcy5$targets object to the reference RNA information present in glasRGcy3$targets object, and viceversa, reconsituting a complete dye-swap pair of hybridizations, as shown below.

We first compared and counted the file names contained in the mapInfo, glasRGcy3$targets, and RobjectglasRGcy5$targets objects, as follows:

```
> ###Compare and count the file names in mapInfo, glasRGcy3$targets, and glasRGcy5$targets
> table(FirstSet=mapInfo$Hybridization %in% glasRGcy3$targets$Array.Data.File,
       SecondSet=mapInfo$Hybridization %in% glasRGcy5$targets$Array.Data.File)

        SecondSet
FirstSet FALSE TRUE
   FALSE     0  162
   TRUE    162    0
```

We then combined the dye-swap mapping information with the phenotypic information for both the **first** and **second** sets of hybridizations, as follows:

```
> ###Combine mapInfo and phenotypic information for the FIRST set of hybridizations
> newTargetsCy3 <- merge(glasRGcy3$targets, mapInfo, sort=FALSE,
                         by.x="Array.Data.File", by.y="Hybridization")
> ###Combine mapInfo and phenotypic information for the SECONDset of hybridizations
> newTargetsCy5 <- merge(glasRGcy5$targets, mapInfo, sort=FALSE,
                         by.x="Array.Data.File", by.y="Hybridization")
```

We subsequently combined together the mapped phenotypic information obtained from both the **first** and **second** sets of hybridizations, as follows:

```
> ###Add phenotypic information from the FIRST to the SECOND set of hybridizations
> newTargetsCy3Cy5 <- merge(newTargetsCy3, newTargetsCy5 , sort=FALSE,
                            by.x="Cy3", by.y="Cy5", suffixes=c(".Cy3", ".Cy5"))
> ###Add phenotypic information from the SECOND to the FIRST set of hybridizations
> newTargetsCy5Cy3 <- merge(newTargetsCy5, newTargetsCy3 , sort=FALSE,
                            by.x="Cy5", by.y="Cy3", suffixes=c(".Cy5", ".Cy3"))
```

We hence simplified these phenotypic tables by keeping only the columns carrying non-redundant information, as follows:

```
> ###Define a function to identify columns carrying non redundant information
> keepRelevant <- function(x) length(unique(x)) >1
> ###Keep only the columns carrying information: FIRST set of hybridizations
> newTargetsCy3Cy5 <- newTargetsCy3Cy5[, apply(newTargetsCy3Cy5, 2, keepRelevant)]
> ###Keep only the columns carrying information: SECOND set of hybridizations
> newTargetsCy5Cy3 <- newTargetsCy5Cy3[, apply(newTargetsCy5Cy3, 2, keepRelevant)]
```

We finally updated the `RGList-Class` instances for both the **first** and **second** sets of hybridizations by adding the mapped phenotypic information, as follows:

```
> ###Assign the merged object newTargetsCy3Cy5 to glasRGcy3: FIRST set of hybridizations
> if (all(glasRGcy3$targets$Array.Data.File == newTargetsCy3Cy5$targets$Array.Data.File.Cy3)) {
         glasRGcy3$targets <- newTargetsCy3Cy5
         print("Updating the target information")
 }

[1] "Updating the target information"

> ###Assign the merged object newTargetsCy5Cy3 to glasRGcy5: SECOND set of hybridizations
> if (all(glasRGcy5$targets$Array.Data.File == newTargetsCy5Cy3$targets$Array.Data.File.Cy5)) {
         glasRGcy5$targets <- newTargetsCy5Cy3
         print("Updating the target information")
 }

[1] "Updating the target information"
```

3.2.2 Gene expression correction and summarization

Once the two sets of dye-swap hybridizations were combined together, we could finally pre-process, normalize, and summarize the gene expression data contained in the `mammaPrintData` package, as described below. To this end we applied the "Rosetta error model" implemented by Weng et al. [14], as described in the original manuscript by Glas and colleagues [3]. This procedure enabled to compute an overall log10 expressio ratio for each microarray feature, using the error-weighted mean calculated over replicated identical probes, as implemented by Weng and colleagues in equation number 16, section 3.1 ("Error-weighted replication combining") [14]:

$$\overline{x} = \frac{\sum_i w_i x_i}{\sum_i w_i}$$

Where:

$$w_i = \frac{1}{\sigma^2_{x_i}}$$

Assuming that x_i is the measured log ratio, and $\sigma^2_{x_i}$ is the measured log ration error, and N the numbed of replicates.

In the present study the log ratio measurements and errors were directly extracted from the raw data files generated by the Agilent Feature Extraction software, and dowloaded from the ArrayExpress data base (see vignette in the `mammaPrintData` package for details). The chunk of `R` code below shows the `correctLogRatio` function, which implements the method above, and was used to correct and summarize the log ratio for each probe on the MammaPrint 1.9k microarray.

```
> ###Function correcting and summarizing log ratios based on tapply() over repeated measurements
> logRatioCorrect <- function(x, y, z) {
        x <- tapply(X=x, INDEX=z, FUN=function(x) x, simplify=FALSE )
        y <- tapply(X=y, INDEX=z, FUN=function(x) x, simplify=FALSE )
        mapply(x, y, FUN=function(x, y) {
                w <- 1 / y^2
                sum(x * w) / sum(w)
        } )
 }
```

The chunk of `R` code below shows the normalization of the **first** set of hybridizations, using probe names to identifiy repeated measurements:

```
> ###Turn log ratios in a numeric matrix and then turn to a data.frame to use mapply()
> logRat <- apply(glasRGcy3$logRatio, 2, as.numeric)
> logRat <- as.data.frame(logRat)
> ###Turn log ratios in a numeric matrix and then turn to a data.frame to use mapply()
> logRatError <- apply(glasRGcy3$logRatioError, 2, as.numeric)
> logRatError <- as.data.frame(logRatError)
> ###Get probe names to be used as INDEX by tapply
> myIndexGenes <- paste(glasRGcy3$genes$Comment.AEReporterName,
                        glasRGcy5$genes$Reporter.Database.Entry.unigene, sep="_")
> myIndexGenes <- gsub("_$", "", myIndexGenes)
> ###Correct and Average overprobe names for the FIRST set of hybridizations
> matCy3 <- mapply(x=logRat, y= logRatError, MoreArgs=list(z=myIndexGenes), FUN=logRatioCorrect)
```

The chunk of `R` code below shows the normalization of the **second** set of hybridizations, using probe names to identifiy repeated measurements:

```
> ###Turn log ratios in a numeric matrix and then turn to a data.frame to use mapply()
> logRat <- apply(glasRGcy5$logRatio, 2, as.numeric)
> logRat <- as.data.frame(logRat)
> ###Turn log ratios in a numeric matrix and then turn to a data.frame to use mapply()
> logRatError <- apply(glasRGcy5$logRatioError, 2, as.numeric)
> logRatError <- as.data.frame(logRatError)
> ###Get probe names to be used as INDEX by tapply
> myIndexGenes <- paste(glasRGcy5$genes$Comment.AEReporterName,
                        glasRGcy5$genes$Reporter.Database.Entry.unigene, sep="_")
> myIndexGenes <- gsub("_$", "", myIndexGenes)
> ###Correct and Average overprobe names for the SECOND set of hybridizations
> matCy5 <- mapply(x=logRat, y= logRatError, MoreArgs=list(z=myIndexGenes), FUN=logRatioCorrect)
```

The chunk of `R` code below was used to generate a non-redundant data.frame containing the annotation for the unique microarray features of the MammaPrint 1.9k microarray (**first** set of hybridizations).

```
> ###Get the unique gene annotation for the FIRST set of hybridizations
> myIndexGenes <- paste(glasRGcy3$genes$Comment.AEReporterName,
                  glasRGcy3$genes$Reporter.Database.Entry.unigene, sep="_")
> myIndexGenes <- gsub("_$", "", myIndexGenes)
> gnsAnnCy3 <- sapply(glasRGcy3$genes, function(x,y) {
         tapply(X=x, INDEX=y, FUN=function(x) x[1] ) },
                    y=myIndexGenes)
> ###Process as data.frame
> gnsAnnCy3 <- as.data.frame(gnsAnnCy3, stringsAsFactors=FALSE)
> gnsAnnCy3$genes231 <- as.logical(gnsAnnCy3$genes231)
> gnsAnnCy3$genes70 <- as.logical(gnsAnnCy3$genes70)
> gnsAnnCy3$gns231Cors <- as.numeric(gnsAnnCy3$gns231Cors)
> ###Remove microarray layout information
> out <- unlist(lapply( c("Row","Column"), grep, colnames(gnsAnnCy3)))
> gnsAnnCy3 <- gnsAnnCy3[, - out]
```

The chunk of R code below was used check the exact correspondence between the rows of the annotation table and the gene expression matrix (**first** set of hybridizations):

```
> ###Check correspondance between annotation and overall expression
> all(rownames(gnsAnnCy3) == rownames(matCy3))

[1] TRUE
```

The chunk of R code below was used to generate a non-redundant data.frame containing the annotation for the unique microarray features of the MammaPrint microarray (**second** set of hybridizations).

```
> ###Get the unique gene annotation for the SECOND set of hybridizations
> myIndexGenes <- paste(glasRGcy5$genes$Comment.AEReporterName,
                  glasRGcy5$genes$Reporter.Database.Entry.unigene, sep="_")
> myIndexGenes <- gsub("_$", "", myIndexGenes)
> gnsAnnCy5 <- sapply(glasRGcy5$genes, function(x,y) {
         tapply(X=x, INDEX=y, FUN=function(x) x[1] ) },
                    y=myIndexGenes)
> ###Process as data.frame
> gnsAnnCy5 <- as.data.frame(gnsAnnCy5, stringsAsFactors=FALSE)
> gnsAnnCy5$genes231 <- as.logical(gnsAnnCy5$genes231)
> gnsAnnCy5$genes70 <- as.logical(gnsAnnCy5$genes70)
> gnsAnnCy5$gns231Cors <- as.numeric(gnsAnnCy5$gns231Cors)
> ###Remove microarray layout information
> out <- unlist(lapply( c("Row","Column"), grep, colnames(gnsAnnCy5)))
> gnsAnnCy5 <- gnsAnnCy5[, - out]
```

The chunk of R code below was used check the exact correspondence between the rows of the annotation table and the gene expression matrix (**second** set of hybridizations):

```
> ###Check correspondance between annotation and overall expression
> all(rownames(gnsAnnCy5) == rownames(matCy5))

[1] TRUE
```

The chunk of R code below was used to compare the non-redundant data.frames corresponding to each set of dye-swap hybridizations and generate a unique final annotation data.frame.

```
> ###Compare the two annotation and keep just one data.frame if identical
> if (all(gnsAnnCy3 == gnsAnnCy5, na.rm=TRUE)) {
         gnsAnn <- gnsAnnCy3
         rm(gnsAnnCy3, gnsAnnCy5)
         print("Creating the final annotation data.frame")
 }

[1] "Creating the final annotation data.frame"
```

The chunk of R code below was used to combine the corrected and summarized log ratios from each set of dye-swap hybridizations in a unique overall measurement. Since the method originally used to combine the two dye-swap sets was not explicitly described in the original manuscript by Glas and colleagues [3], we used the arithmetic mean between one set of measurements and the inverse of the other set. The final summarized ratio will express the relative change in expression between cancer (numerator) and the reference RNA (denominator). In the following R code chunk multiplying by -1 the log ratio gene expression matrix is equivalent to flippling it. Note that we inverted the matCy3 matrix, since it corresponded to the dye-swap set in which cancer samples were labeled with Cy3.

```
> ###Combine the summarized log ratio from each set of hybridizarions
> ###Note that we are flipping matCy3
> matCy3Cy5 <- (-1*matCy3 + matCy5)/2
```

The chunks of R code below were used to assemble all processed phenotypic information, the normalized and summarized gene expression data, and the non-redundant gene annotation information into an ExpressionSet instance for later use in all our analyses.

Creation of a MIAME instance to store the experiment and study information:

```
> ###Require Biobase
> require(Biobase)
> ###Retrieve the phenotypic information from one of the RGList intances
> phenoGlas <- glasRGcy3$targets
> ###Create a MIAME instance
> miame <- new("MIAME", name="GlasCohort",
             pubMedIds="17074082",
             title="ArrayExpress E-TABM-115: corrected log fold-change",
             lab="Agendia",
             url="http://www.ebi.ac.uk/arrayexpress/browse.html?keywords=E-TABM-115")
```

Creation of an AnnotatedDataFrame instance to store the phenotypic information:

```
> ###AnnotatedDataFrame for phenotypic data
> if (all( phenoGlas$Scan.Name.Cy3 == colnames(matCy3Cy5))) {
        rownames(phenoGlas) <- colnames(matCy3Cy5)
        phenoGlasData <- new("AnnotatedDataFrame", phenoGlas)
        print("Creating new AnnotatedDataFrame for phenotypic information")
 } else {
        print("Check sample order of phenotypic information")
 }

[1] "Creating new AnnotatedDataFrame for phenotypic information"
```

Creation of an AnnotatedDataFrame instance to store the annotation information:

```
> ###AnnotatedDataFrame for annotation data
> gnsAnn <- as.data.frame(gnsAnn, stringsAsFactors=FALSE)
> featureData <- new("AnnotatedDataFrame", gnsAnn)
```

Creation of the final glasEset ExpressionSet instance:

```
> ###Create the ExpressionSet
> glasEset <- new("ExpressionSet",
                phenoData = phenoGlasData, featureData = featureData,
                experimentData = miame, exprs = matCy3Cy5)
> ###Save for later use
> save(glasEset, file="objs/glasEset.rda")
```

The glasEset ExpressionSet instance above is available from http://luigimarchionni.org/breastTSP.html. Furthermore, such processed gene expression data is also provided NA the BMC Genomics web-

site.

3.3 Buyse cohort: ArrayExpress "E-TABM-77" series

The Buyse data set [4] comprises patients from an European multicenter study performed to independently validate the MammaPrint assay. This cohort accounted a total of 307 patients, for 302 of which the complete clinical information was available, and were therefore considered in the comparative analyses with the Adjuvant! Online software [15]. Similarly to the Glas cohort also in this case a dye-swap two-colors design was adopted, comparing each sample against the same reference RNA (307 samples * 2 channels * 2 dye-swap hybridizations = 1228 total data points). Below is shown the `R` code used to load the two Buyse cohort `RGList-class` instances contained in the `mammaPrintData` package, one for each dye-swap set of hybridizations.

```
> ###Load the mammaPrintData library
> require(mammaPrintData)
> ###Load the Buyse cohort data
> data(buyseRG)
> ###Dimensions of the RGList objects
> dim(buyseRGcy5)

[1] 1900  307

> dim(buyseRGcy3)

[1] 1900  307
```

3.3.1 Gene expression correction and summarization

As described in details in the `mammaPrintData` package vignette, unlike the case of the "E-TABM-115" series, the phenotypic information associated with the "E-TABM-77" ArrayExpress record available on Apr 08, 2013 was complete. The SDRF annotation table, in fact, accounted for 1228 total rows, one for each hybridization channel (307 samples for 2 channels, for 2 replicated dye-swap hybridizations). Based on this information it was possible to associate the clinical information for **all** the hybridizations that were performed withouth the need of any additional mapping information.

The chunk of `R` code below shows the normalization of the **first** set of hybridization, using probe names to identifiy repeated measurements:

```
> ###Turn log ratios in a numeric matrix and turn to data.frame
> logRat <- apply(buyseRGcy3$logRatio, 2, as.numeric)
> logRat <- as.data.frame(logRat)
> ###Turn log ratio errors in a numeric matrix and turn to data.frame
> logRatError <- apply(buyseRGcy3$logRatioError, 2, as.numeric)
> logRatError <- as.data.frame(logRatError)
> ###Get probe names to be used as INDEX by tapply
> myIndexGenes <- paste(buyseRGcy3$genes$Comment.AEReporterName,
                      buyseRGcy3$genes$Reporter.Database.Entry.unigene, sep="_")
> myIndexGenes <- gsub("_$", "", myIndexGenes)
> ###Correct and Average overprobe names for the FIRST set of hybridizations
> matCy3 <- mapply(x=logRat, y= logRatError, MoreArgs=list(z=myIndexGenes), FUN=logRatioCorrect)
```

The chunk of `R` code below shows the normalization of the **second** set of hybridization, using probe names to identifiy repeated measurements:

```
> ###Turn log ratios in a numeric matrix and turn to data.frame
> logRat <- apply(buyseRGcy5$logRatio, 2, as.numeric)
> logRat <- as.data.frame(logRat)
> ###Turn log ratio errors in a numeric matrix and turn to data.frame
> logRatError <- apply(buyseRGcy5$logRatioError, 2, as.numeric)
> logRatError <- as.data.frame(logRatError)
> ###Get probe names to be used as INDEX by tapply
> myIndexGenes <- paste(buyseRGcy5$genes$Comment.AEReporterName,
                       buyseRGcy5$genes$Reporter.Database.Entry.unigene, sep="_")
> myIndexGenes <- gsub("_$", "", myIndexGenes)
> ###Correct and Average overprobe names for the SECOND set of hybridizations
> matCy5 <- mapply(x=logRat, y= logRatError, MoreArgs=list(z=myIndexGenes), FUN=logRatioCorrect)
```

The chunk of R code below was used to generate a non-redundant data.frame containing the annotation for the unique microarray features of the MammaPrint microarray (**first** set of hybridizations).

```
> ###Get the unique gene annotation for the FIRST set of hybridizations
> myIndexGenes <- paste(buyseRGcy3$genes$Comment.AEReporterName,
                  buyseRGcy3$genes$Reporter.Database.Entry.unigene, sep="_")
> myIndexGenes <- gsub("_$", "", myIndexGenes)
> gnsAnnCy3 <- sapply(buyseRGcy3$genes,  function(x,y) {
        tapply(X=x, INDEX=y, FUN=function(x) x[1] ) },
                   y=myIndexGenes)
> ###Process as data.frame
> gnsAnnCy3 <- as.data.frame(gnsAnnCy3, stringsAsFactors=FALSE)
> gnsAnnCy3$genes231 <- as.logical(gnsAnnCy3$genes231)
> gnsAnnCy3$genes70 <- as.logical(gnsAnnCy3$genes70)
> gnsAnnCy3$gns231Cors <- as.numeric(gnsAnnCy3$gns231Cors)
> ###Remove microarray layout information
> out <- unlist(lapply( c("Row","Column"), grep, colnames(gnsAnnCy3)))
> gnsAnnCy3 <- gnsAnnCy3[, - out]
> ###Check correspondance between annotation and overall expression
> all(rownames(gnsAnnCy3) == rownames(matCy3))

[1] TRUE
```

The chunk of R code below was used to generate a non-redundant data.frame containing the annotation for the unique microarray features of the MammaPrint microarray (**second** set of hybridizations).

```
> ###Get the unique gene annotation for the SECOND set of hybridizations
> myIndexGenes <- paste(buyseRGcy5$genes$Comment.AEReporterName,
                  buyseRGcy5$genes$Reporter.Database.Entry.unigene, sep="_")
> myIndexGenes <- gsub("_$", "", myIndexGenes)
> gnsAnnCy5 <- sapply(buyseRGcy5$genes,  function(x,y) {
        tapply(X=x, INDEX=y, FUN=function(x) x[1] ) },
#                   y=buyseRGcy5$genes$Comment.AEReporterName)
                   y=myIndexGenes)
> ###Process as data.frame
> gnsAnnCy5 <- as.data.frame(gnsAnnCy5, stringsAsFactors=FALSE)
> gnsAnnCy5$genes231 <- as.logical(gnsAnnCy5$genes231)
> gnsAnnCy5$genes70 <- as.logical(gnsAnnCy5$genes70)
> gnsAnnCy5$gns231Cors <- as.numeric(gnsAnnCy5$gns231Cors)
> ###Remove microarray layout information
> out <- unlist(lapply( c("Row","Column"), grep, colnames(gnsAnnCy5)))
> gnsAnnCy5 <- gnsAnnCy5[, - out]
> ###Check correspondance between annotation and overall expression
> all(rownames(gnsAnnCy5) == rownames(matCy5))

[1] TRUE
```

The chunk of R code below was used to compare the non-redundant data.frames corresponding to each set of

dye-swap hybridizations and generate a unique final annotation data.frame.

```
> ###Compare the two annotation and keep just one data.frame if identical
> if (all(gnsAnnCy3 == gnsAnnCy5, na.rm=TRUE)) {
         gnsAnn <- gnsAnnCy3
         rm(gnsAnnCy3, gnsAnnCy5)
         print("Creating the final annotation data.frame")
 }

[1] "Creating the final annotation data.frame"
```

The chunk of R code below was used to combine the corrected and summarized log ratios from each set of dye-swap hybridizations in a unique overall measurement. Since the method originally used to combine the two sets was not explicitly described in the original manuscript by Buyse and colleagues [4], also in this case we used the arithmetic mean between one set of measurements and the inverse of the other set. The final summarized ratio will express the relative change in expression between cancer (numerator) and the reference RNA (denominator). In the following R code chunk multiplying by -1 the log ratio gene expression matrix is equivalent to flippling it. Note that we inverted the matCy5 matrix, since it corresponded to the dye-swap set in which cancer samples were labeled with Cy3.

```
> ###Combine the summarized log ratio from each set of hybridizarions
> ###Note we are flipping matCy5
> matCy3Cy5 <- (-1*matCy5 + matCy3)/2
```

The chunks of R code below were used to assemble all processed phenotypic information, the normalized and summarized gene expression data, and the non-redundant gene annotation information into an ExpressionSet instance for later use in all our analyses.

To this end we firstly assembled a unique phenotypic information table by comparing and then combining the information associated with each dye-swap set of hybrizations, as shown below.

```
> ###Check sample correspondance beween the two hybridization sets
> all( buyseRGcy5$targets$Cy3 == buyseRGcy3$targets$Cy5 )

[1] TRUE

> ###Combine phenotypic information from the two RGList intances
> phenoBuyse <- cbind(stringsAsFactors=FALSE, Patients=buyseRGcy3$targets$Cy5,
               buyseRGcy3$targets[!buyseRGcy3$targets %in% buyseRGcy5$targets])
> phenoBuyse <- merge(phenoBuyse, buyseRGcy5$targets, by.x="Patients", by.y="Cy3", sort=FALSE)
```

Creation of a MIAME instance to store the experiment and study information:

```
> ###Require Biobase
> require(Biobase)
> ###Create a MIAME instance
> miame <- new("MIAME", name="BuyseCohort",
              pubMedIds="16954471",
              title="ArrayExpress:E-TABM-77; corrected log fold-change",
              lab="Agendia",
              url="http://www.ebi.ac.uk/arrayexpress/browse.html?keywords=E-TABM-77")
```

Creation of an AnnotatedDataFrame instance to store the phenotypic information:

```
> ###AnnotatedDataFrame for phenotypic data
> if (all( phenoBuyse$Scan.Name.Cy5 == colnames(matCy3Cy5))) {
         rownames(phenoBuyse) <- colnames(matCy3Cy5)
         phenoBuyseData <- new("AnnotatedDataFrame", phenoBuyse)
 }
```

Creation of an AnnotatedDataFrame instance to store the annotation information:

```
> ###AnnotatedDataFrame for annotation data
> gnsAnn <- as.data.frame(gnsAnn, stringsAsFactors=FALSE)
> featureData <- new("AnnotatedDataFrame", gnsAnn)
```

Creation of the final `buyseEset` `ExpressionSet` instance:

```
> ###Create the ExpressionSet
> buyseEset <- new("ExpressionSet",
                  phenoData = phenoBuyseData, featureData = featureData,
                  experimentData = miame, exprs = matCy3Cy5)
> ###Save for later use
> save(buyseEset, file="objs/buyseEset.rda")
```

The `buyseEset` ExpressionSet instance above is available from `http://luigimarchionni.org/breastTSP.html`. Furthermore, such processed gene expression data is also provided NA the BMC Genomics website.

3.4 End-point and prognostic groups selection

In the present study we have subset the gene expression data limiting our investigation to the 70-gene signature only. Furthermore, similarly to what was done in the original study by van't Veer and colleagues [1], we have defined patients prognostic groups using the time to development of a distant metastasis as first recurrence event as follows:

- "Low-risk" group: patients who remained disease free for at least 5 years;
- "High-risk" group: patients who developed a distant metastases within 5 years;

3.4.1 Training set: Glas cohort (E-TABM-115)

The complete Glas data set was originally used to implement the MammaPrint assay re-developing the 70-gene prognostic signature on the new 1.9k microarray platform. This cohort accounts for a total 162 lymphnode negative patients, including the 78 cases originally analyzed in the van't Veer study [1] to develop the prognostic signature, and additional patients from the Van de Vijver [2] study. We have used this cohort to train and validate novel TSP-based prognostic predictors, reproducing the approach used in the original study. In the following `R` code chunks is shown how to identify the microarray features corresponding to the 70-gene signature, and select the cases to be used in the analyses.

Load the previously saved `ExpressionSet` instance for the Glas cohort:

```
> ###Load normalized and summarized data from Glas cohort
> load("objs/glasEset.rda")
```

Such processed gene expression data is also provided as "Additional data" within the compressed archive available from the BMC Genomics website. Alternatively the processed gene expression data obtained can be also dowloaded from the website accompanying this manuscript, as follows:

```
> ###Define the url for the ExpressionSet file from the website
> url <- "http://luigimarchionni.org/breastTSP/glasEset.rda"
> ###Loadthe ExpressionSet file from the website
> load(url)
```

Data set summary:

```
> ###Dimension of the ExpressionSet
> dim(glasEset)

Features  Samples
    1149      162

> ###Count metastases event by original data sets
> table(DataSet = pData(glasEset)$putativeCohort.Cy5,
       MetastaticEvent = pData(glasEset)$FiveYearMetastasis)

                     MetastaticEvent
DataSet               FALSE TRUE
  putativeVantVeer       44   34
  putativeVanDeVijver    72   12
```

The 70-gene prognostic signature comprises the top 70 microarray features showing the highest absolute correlation with survival, as described in the supplemenatary section of the original van't Veer study (Table S2) [1]. However, out of these 70 genes only 69 could be mapped on the MammaPrint 1.9k platform using the feature identifiers provided, as described in the `mammaPrintData` package vignette and showed below.

```
> ###Counting the 70-gene microarray features available on the new platform
> table(featureData(glasEset)$genes70)

FALSE  TRUE
 1079    70

> ###Create a logical vector to select the 70-gene prognostic signature genes
> select70geneGlas <- featureData(glasEset)$genes70
```

Subset the gene expression data limiting to the 70-gene microarray features:

```
> ###Subset the ExpressionSet: 70-gene signature only and all patients
> glasDataAll <- glasEset[select70geneGlas, ]
```

Identification and selection of the patients of the Glas combined cohort (van't Veer and Van de Vijver cases) who developed a distant metastases as first recurrence event within five years, and of the patients who remained disease free for at least five years.

```
> ###Subset the ExpressionSet: cases with metastasis within 5 years
> ###or disease free for at least 5 years
> glasCasesAll.ttm <- which( ! is.na(pData(glasDataAll)$FiveYearMetastasis) & !
                           (pData(glasDataAll)$OS < 5 & pData(glasDataAll)$TTMevent == 0) )
> glasDataAll.ttm <- glasDataAll[, glasCasesAll.ttm]
> ###Define "Low-risk" and "High-risk" prognostic groups
> glasGroupAll.ttm <- (pData(glasDataAll.ttm)$FiveYearMetastasis)
> ###Set "Low-risk" == 1 and "High-risk" == 0
> glasGroupAll.ttm <- (! glasGroupAll.ttm) * 1
```

The recurrence status summary for the Glas combined cohort is shown below. Four patients who did not develop metastases and for which the reported overal survival (OS) was shorter than 5 years were excluded from further analyses.

```
> ###Data set summary
> summary(pData(glasDataAll)$FiveYearMetastasis)

   Mode   FALSE    TRUE    NA's
logical     116      46       0

> ###Dimentions of the test set
> dim(glasDataAll.ttm)

Features  Samples
      70      158
```

```
> ###Excluded patients
> table(Metastasis = pData(glasDataAll)$FiveYearMetastasis,
       "OS < 5" = pData(glasDataAll)$OS < 5 & pData(glasDataAll)$TTMevent == 0)

          OS < 5
Metastasis FALSE TRUE
     FALSE   112    4
     TRUE     46    0

> ###Count "High-risk" and "Low-risk" patients in the test set
> table(glasGroupAll.ttm)

glasGroupAll.ttm
  0   1
 46 112
```

3.4.2 Test set: Buyse cohort (E-TABM-77)

The Buyse data set [4] comprises a total of 307 patients from a European multicenter study performed to independently validate the MammaPrint assay. We therefore used this cohort to validate the novel TSP-based prognostic predictors we developed using the Glas cohort (see above). In the following R code chunks is shown how to identify the microarray features corresponding to the 70-gene signature, and select the cases to be used in the analyses.

```
> ###Load normalized and summarized data from Buyse cohort
> load("objs/buyseEset.rda")  #or load("buyseEsetFromUrl.rda")
```

Such processed gene expression data is also provided as "Additional data" within the compressed archive available from the BMC Genomics website. Alternatively the processed gene expression data obtained can be also dowloaded from the website accompanying this manuscript, as follows:

```
> ###Define the url for the ExpressionSet file from the website
> url <- "http://luigimarchionni.org/breastTSP/buyseEset.rda"
> ###Loadthe ExpressionSet file from the website
> load(url)
```

Data set summary:

```
> ###Dimension of the ExpressionSet
> dim(buyseEset)

Features  Samples
    1149      307

> ###Count metastases events
> table(MetastaticEvent = pData(buyseEset)$FiveYearRecurrence)

MetastaticEvent
FALSE  TRUE
  260    47
```

Identification of the microarray features corresponding to the 70-gene prognostic signature mapped on the MammaPrint 1.9k platform:

```
> ###Counting the 70-gene microarray features available on the new platform
> table(featureData(buyseEset)$genes70)

FALSE  TRUE
 1079    70
```

```
> ###Logical vector to select the 70-gene prognostic signature genes
> select70geneBuyse <- featureData(buyseEset)$genes70
```

Subset the gene expression data limiting to the 70-gene microarray features:

```
> ###Subset the ExpressionSet: 70-gene signature only and all patients
> buyseDataAll <- buyseEset[select70geneBuyse, ]
```

Identification and selection of the patients of the Buyse cohort developed a distant metastases as first recurrence event within five years, and of the patients who remained disease free for at least five years.

```
> ###Subset the ExpressionSet: all cases
> buyseCasesAll.ttm <- which( ! is.na(pData(buyseDataAll)$FiveYearRecurrence) )
> buyseDataAll.ttm <- buyseDataAll[, buyseCasesAll.ttm]
> ###Define "High-risk" and "Low-risk" prognostic groups
> buyseGroupAll.ttm <- (pData(buyseDataAll.ttm)$FiveYearRecurrence)
> ###Set "Low-risk" == 1 and "High-risk" == 0
> buyseGroupAll.ttm <- (! buyseGroupAll.ttm) * 1
```

The recurrence status summary for the Buyse cohort is shown below.

```
> ###Data set summary
> summary(pData(buyseDataAll)$FiveYearRecurrence)

   Mode   FALSE    TRUE    NA's 
logical     260      47       0 

> ###Dimentions of the test set
> dim(buyseDataAll.ttm)

Features  Samples 
      70      307 

> ###Count "Low-risk" and "High-risk" patients in the test set
> table(buyseGroupAll.ttm)

buyseGroupAll.ttm
  0   1 
 47 260 
```

3.5 Comparing the clinical information across studies and cohorts

We have compared the clinical information available for the Glas cohort with the one available for the original van't Veer and Van de Vijver cohorts, since the information provided with the "E-TABM-115" ArrayExpress [8] series did not explicitly identify Glas data set samples membership to the original cohorts. To this end we have assembled and used the `seventyGeneData` `R-Bioconductor` package, the content of which is shown in Table 2.

This package will be made with the next `Bioconductor` release and can be currently installed from the website accompanying the manuscript as follows:

```
> ###Obtain and install the data package from the manuscript website
> marchionniRepos <- "http://luigimarchionni.org/software/"
> install.packages("seventyGeneData", repos=marchionniRepos)
```

Table 2: The seventyGeneData package content

Package	Item	Title
seventyGeneData	vanDeVijver	Gene expression, annotations and clinical information for the Van de Vijver cohort
seventyGeneData	vantVeer	Gene expression, annotations and clinical information for the van't Veer cohort

The chunk of `R` code below shows how to load the `ExpressionSet` objects for the van't Veer and Van de Vijver cohorts from the `seventyGeneData` package:

```
> ###Load data from the van't Veer et al study
> data(vantVeer)
> data(vanDeVijver)
> ###Dimensions of the  vantVeer and vanDeVijver ExpressionSets
> dim(vantVeer)

Features  Samples
   24481      117

> dim(vanDeVijver)

Features  Samples
   24496      295
```

3.5.1 The van't Veer cases in the Glas study

Below is the code to compare the phenotypic information for putative van't Veer cases contained in the Glas cohort to the information originally provided (for detailed informarmation see the `mammaPrintData` package vignette). We firstly extracted the phenotypic information from the `glasDataAll` and `vantVeer` objects.

```
> ###Get phenotypes for putative van't Veer cases from the Glas study
> pGlasVtV <- pData(glasDataAll)[pData(glasDataAll)$putativeCohort.Cy5 == "putativeVantVeer", ]
> dim(pGlasVtV)

[1] 78 27

> ###Get phenotypes for van't Veer cases from the original study (BRCA cases excluded)
> pVtV <- pData(vantVeer)[pData(vantVeer)$DataSetType != "BRCA",]
> dim(pVtV)

[1] 97 16
```

We subsequently compared such clinical and phenotypic information, including node status information, and time to development of a distant metastasis, for the cases in common.

```
> ###Is the time to development of metastasis identical for the common cases?
> all(pGlasVtV$TTM %in% round(pVtV$TTM,2))

[1] FALSE

> ###For which patients in the Glas study the time to metastases is different?
> checkGlasVtV <- which(! pGlasVtV$TTM %in% round(pVtV$TTM,2))
> ###For which patients in the van't Veer study the time to metastases is different?
> checkVtV <- which( (!round(pVtV$TTM,2) %in% pGlasVtV$TTM)
                  & pVtV$DataSetType != "19samples")
```

In Table 3 below is shown the clinical information contained in the Glas study for the case with **different** time to development of a metastasis as first recurrence event:

Table 3: Cases from the Glas study missing or with different clinical information in the van't Veer study

Sample.Name.Cy5	TTM	TTMevent	OS	OSevent
1181311	8.37	0.00	8.37	0.00
1118611	8.42	0.00	8.42	0.00
1181623	8.65	0.00	8.65	0.00
1193921	9.12	1.00	9.92	0.00
1181621	9.41	0.00	9.41	0.00
1197111	10.02	0.00	10.02	0.00
1118613	11.36	0.00	11.36	0.00
1194323	12.74	0.00	12.74	0.00
1197113	12.77	0.00	12.77	0.00
1181413	14.26	0.00	14.26	0.00
1181411	14.82	0.00	14.82	0.00
1181323	15.35	0.00	15.35	0.00

In Table 4 is shown the clinical information contained in the van't Veer study for the case with **different** time to development of a metastasis as first recurrence event:

Table 4: Cases from the van't Veer study missing or with different clinical information in the Glas study

SampleName	DataSetType	TTM	TTMevent	followup.time.yr	metastases	age	Brca1.mutation
Sample 20	greater_than_5y	5.10	0	5.10	0	34	0
Sample 15	greater_than_5y	5.23	0	5.23	0	46	0
Sample 19	greater_than_5y	5.72	0	5.72	0	48	0
Sample 2	greater_than_5y	6.44	0	6.44	0	44	0
Sample 18	greater_than_5y	7.41	0	7.41	0	32	0
Sample 16	greater_than_5y	8.39	0	8.39	0	49	0
Sample 21	greater_than_5y	10.54	0	10.54	0	39	0
Sample 3	greater_than_5y	10.66	0	10.66	0	41	0
Sample 33	greater_than_5y	11.46	0	11.46	0	52	0
Sample 5	greater_than_5y	11.98	0	11.98	0	48	0
Sample 1	greater_than_5y	12.53	0	12.53	0	43	0
Sample 25	greater_than_5y	13.42	0	13.42	0	45	0

As revealed by the comparison of Tables 3 and 4 above, the patients with different phenotypic information were all **metastasis free** at the time of the original van't Veer study, and showed **longer** time to of metastasis development in the Glas data set. This is therefore compatible with an updated follow-up available for the Glas study that was performed at a later time.

3.5.2 The Van de Vijver cases in the Glas study

Below is the `R` code used to compare the phenotypic information for the putative Van de Vijver cases contained in the Glas cohort to the information originally provided (for detailed informarmation see the `mammaPrintData` package vignette). To this end we first extracted the sample identifiers and the phenotypic information from the `glasDataAll` and `vanDeVijver` objects.

```
> ###Get phenotypes for putative Van de Vijver cases from the Glas study
> pGlasVDV <- pData(glasDataAll)[pData(glasDataAll)$putativeCohort.Cy5 == "putativeVanDeVijver", ]
> ###Extract the putative identifiers for the Van de Vijver cases
> glasVanDeViverIds <- as.numeric(gsub(".+_", "",
                                  pGlasVDV$Scan.Name.Cy5[pGlasVDV$putativeCohort.Cy5
                                                         == "putativeVanDeVijver"]))
> ###Note that ID 397 is repeated twice
> any(duplicated(glasVanDeViverIds))
```

```
[1] TRUE

> glasVanDeViverIds[which(duplicated(glasVanDeViverIds))]

[1] 397

> ###Extract phenotypes for the putative Van de Vijver cases from the Glas study
> sel <- !duplicated(glasVanDeViverIds)
> pGlasVDV <- pGlasVDV[sel,]
> dim(pGlasVDV)

[1] 83 27

> glasVanDeViverIds <- glasVanDeViverIds[sel]
> ###Get phenotypes for Van de Vijver cases from the study
> pVDVneg <- pData(vanDeVijver)[pData(vanDeVijver)$SampleID %in% glasVanDeViverIds,]
> dim(pVDVneg)

[1] 83 18

> ###Reorder by Identifiers
> pGlasVDV <- pGlasVDV[order(glasVanDeViverIds),]
> pVDVneg <- pVDVneg[order(pVDVneg$SampleID),]
```

We subsequently compared the clinical and phenotypic information, including node status information, overall survival, and time to development of a distant metastasis, for the common cases.

```
> ###Is the overall survival identical for the common cases?
> all(pGlasVDV$OS == round(pVDVneg$OS,2))

[1] TRUE

> ###Is the time to development of metastasis identical for the common cases?
> all(pGlasVDV$TTM == round(pVDVneg$TTM,2))

[1] TRUE

> ###Is the overall survival events identical for the common cases?
> all(pGlasVDV$OSevent == pVDVneg$OSevent)

[1] TRUE

> ###Is the time to development of metastasis identical for the common cases?
> all(pGlasVDV$TTMevent == pVDVneg$TTMevent)

[1] TRUE

> ###Count node status for patients from the Van de Vijver cohort
> table(pVDVneg$Posnodes)

 n
83
```

As revealed by the output from the R code chunks above the clinical information provided with the Van de Vijver and Glas studies is identical.

4 Prognostic predictor training

We have applied the Top-Scoring-Pair (TSP) algorithm [5, 6] to predict breast cancer prognosis using the time to development of a metastasis as first recurrence event as our primary end-point. In particular we have applied an extension of the TSP algorithm, the *k*-TSP method, which combines multiple TSP in a unique

classifier. To this end we have employed the Glas and Buyse cohorts [3, 4], which were respectively used to implement and validate the MammaPrint assay, as described below.

4.1 *K*-TSP classifier development using the Glas data set

For training purposes we have applied the *k*-TSP algorithm on the Glas data set [3], selecting the same cases and using the same end-point of the original van't Veer study [1]. In particular we have performed the following analytical tasks:

1. Identification of the complete set of disjoint TSPs predicting recurrence using only the 70-gene signature features.
2. Outcome prediction in the training set using combinations of an increasing k number of TSP.
3. Receiver Operating characteristic (ROC) curve analysis to select the minimal *k*-TSP combination achieving the best prediction, as assessed by the Area Under Curve (AUC).
4. Selection of the best *k*-TSP combination threshold to achieve at best sensitivity (possibly equal to 1), and the best possible specificity;

It should be noted that the choice of restricting the learning process to the 70-gene signature is justified by the fact that the other features present on the MammaPrint microarray are "housekeeeing" genes used for normalization purposes, which do not change across samples and therefore do not carry any prognostic information. Furthermore it must be also noted that for tuning model complexity we have used resubstitution AUC, as opposed to a cross-validated AUC. This apporach works reasonably well for the TSP method, which is not very prone to over-fitting, but might produce over-fitted and inferior prediction models for other algorithms.

4.1.1 Definition of the *k*-TSP classifier training functions

The following `R` code chunks were used to train and test novel *k*-TSP prognostic classifiers, using the van't Veer breast cancer cases of the Glas cohort and the 70-gene prognostic signature microarray features.

Loading the necessary `R` libraries:

```
> ###Require the switchBox library
> require(switchBox)
> ###Require the caret library
> require(caret)
> ###Require the pROC library
> require(pROC)
```

The following `R` code chunk was use to define a function for computing *k*-TSP classification performance results:

```
> ###Function to compute KSP classification performance using the caret library
> getKTSPperformance <- function(data, ktsp, group, ...) {
        out <- table(KTSP.Classify(data, ktsp, ...), group)
        out <- confusionMatrix(out)
        out$overallAccuracy <- mean(out$byClass[c("Sensitivity", "Specificity")])
        return(out)
        }
```

The following `R` code chunk was use to define a function for computing *k*-TSP classification ROC curves:

```
> ###Function to compute ROC and AUC for a k-TSP combination using the pROC library
> getKTSPauc <- function(k, data, group, thrMethod="local maximas",
                         ret=c("threshold", "sens", "spec", "npv", "ppv", "accuracy"), ...) {
        ktsp <- KTSP.Train(data, group, k)
        pred <- KTSP.Classify(data, ktsp, combineFunc = sum)
        perf <- roc(!group, pred, ci=FALSE, plot=FALSE)
        stats <- coords(perf, x=thrMethod, ret=ret, ...)
        out <- list(auc=perf$auc, stats=stats)
 }
```

The following `R` code chunk was use to define a function for plotting k-TSP classification AUC curves:

```
> ###Function to plot AUC classification performance using the pROC library
> plotKTSPauc <- function(k, data, group, ci=TRUE, ...) {
        ktsp <- KTSP.Train(data, group, k)
        pred<- KTSP.Classify(data, ktsp, combineFunc = sum)
        rocOut <- roc(!group, pred, ...)
 }
```

The following chunks of `R` code were used to train the k-TSP classifier using the van't Veer cases from the Glas cohort, the 70-gene set, and the occurrence of a metastasis within five years as end-point. Furthermore below is shown how to compute ROC parameters for distinct combinations of increasing number of TSP.

```
> ###Create a vector to Select the van'tVeer cases contained in the Glas cohort
> sel <- pData(glasDataAll.ttm)$putativeCohort.Cy5 %in% c("putativeVantVeer")
> ###Create an named index vector for the TSP that can be obtained using only the 70-gene signature
> k <- 2:35
> names(k) <- paste("k", k, sep="")
> ###Computing LOCAL MAXIMAS ROC parameters for increasing k-TSP combinations
> rocGlasLocalMax <- lapply(X=k, FUN=getKTSPauc, data=exprs(glasDataAll.ttm)[, sel],
                            group=glasGroupAll.ttm[sel])
> ###Extract AUC information
> glasBestAuc <- sapply(rocGlasLocalMax, function(x) x$auc)
```

4.1.2 *k* optimization based on resubstitution AUC

In Figure 1 AUC values from re-substitution for time to metastasis prediction using combinations of 2 to 35 TSPs.

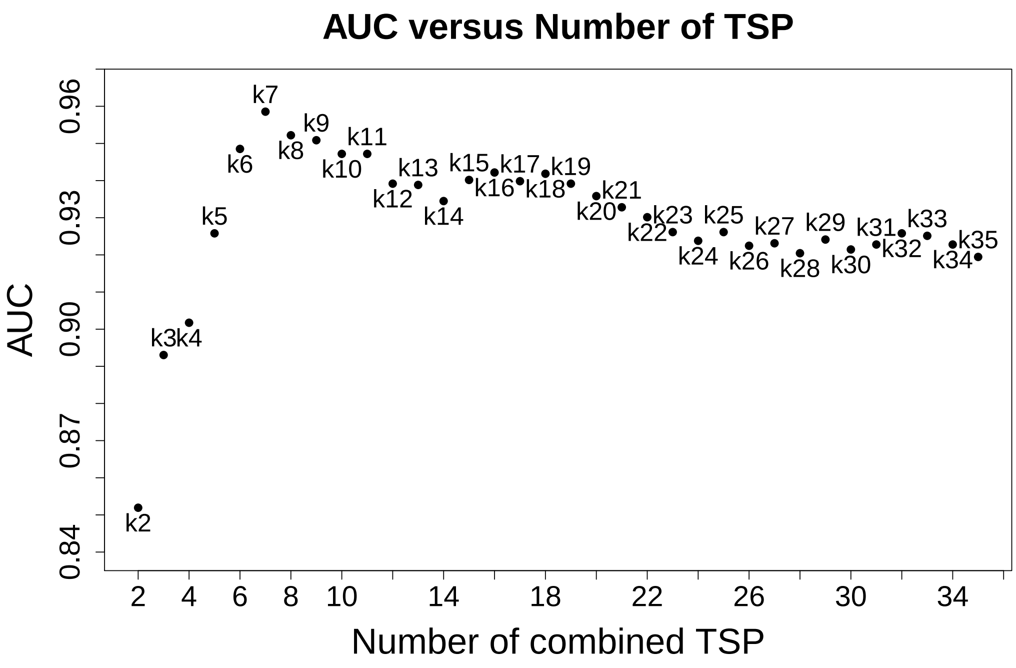

Figure 1: Area Under the Curve (AUC) from re-substitution for combining increasing numbers of TSPs in the training set. AUC is shown on the y-axis, number of pairs k is shown on x-axis.

As shown in Figure 1 the AUC substantially increased by combining from 2 to 6-10 TSPs, while no further improvement was achieved by adding additional TSPs to the predictive algorithm. We therefore focused our further investigation on combinations of 6 to 10 TSPs, with the goal of identifying the **smallest** combination of TSPs achieving 100% sensitivity and the best specificity in the training set.

```
> ###Selection of the best and smallest k-TSP combination in terms of AUC
> goodK <- paste("k", 2:10, sep="")
> ###Extract ROC parameter for the selected k-TSP classifiers
> rocParams <- sapply(rocGlasLocalMax[goodK], function(x) x$stats)
```

In Table 5 are shown the ROC parameters computed on the training set using the k-TSP classifier combining the top first k=5 TSPs:

Table 5: ROC parameters for the k-TSP classifier combining the top 5 TSPs

	sensitivity	specificity	npv	ppv	accuracy
Threshold: 0.500	1.000	0.409	1.000	0.567	0.667
Threshold: 1.500	0.971	0.705	0.969	0.717	0.821
Threshold: 2.500	0.912	0.886	0.929	0.861	0.897
Threshold: 3.500	0.706	0.909	0.800	0.857	0.821
Threshold: 4.500	0.265	0.977	0.632	0.900	0.667
Threshold: Inf	0.000	1.000	0.564	NaN	0.564

In Table 6 are shown the ROC parameters computed on the training set using the k-TSP classifier combining the top first k=6 TSPs:

Table 6: ROC parameters for the *k*-TSP classifier combining the top 6 TSPs

	sensitivity	specificity	npv	ppv	accuracy
Threshold: 1.500	1.000	0.682	1.000	0.708	0.821
Threshold: 2.500	0.912	0.864	0.927	0.838	0.885
Threshold: 3.500	0.853	0.909	0.889	0.879	0.885
Threshold: 4.500	0.471	0.977	0.705	0.941	0.756
Threshold: 5.500	0.206	1.000	0.620	1.000	0.654

In Table 7 are shown the ROC parameters computed on the training set using the *k*-TSP classifier combining the top first k=7 TSPs:

Table 7: ROC parameters for the *k*-TSP classifier combining the top 7 TSPs

	sensitivity	specificity	npv	ppv	accuracy
Threshold: 1.500	1.000	0.659	1.000	0.694	0.808
Threshold: 2.500	0.971	0.864	0.974	0.846	0.910
Threshold: 3.500	0.853	0.909	0.889	0.879	0.885
Threshold: 4.500	0.618	0.977	0.768	0.955	0.821
Threshold: 6.500	0.147	1.000	0.603	1.000	0.628

In Table 8 are shown the ROC parameters computed on the training set using the *k*-TSP classifier combining the top first k=8 TSPs:

Table 8: ROC parameters for the *k*-TSP classifier combining the top 8 TSPs

	sensitivity	specificity	npv	ppv	accuracy
Threshold: 2.500	1.000	0.818	1.000	0.810	0.897
Threshold: 3.500	0.882	0.864	0.905	0.833	0.872
Threshold: 4.500	0.735	0.932	0.820	0.893	0.846
Threshold: 5.500	0.588	0.977	0.754	0.952	0.808
Threshold: 7.500	0.147	1.000	0.603	1.000	0.628

In Table 9 are shown the ROC parameters computed on the training set using the *k*-TSP classifier combining the top first k=9 TSPs:

Table 9: ROC parameters for the *k*-TSP classifier combining the top 9 TSPs

	sensitivity	specificity	npv	ppv	accuracy
Threshold: 2.500	1.000	0.773	1.000	0.773	0.872
Threshold: 3.500	0.912	0.864	0.927	0.838	0.885
Threshold: 4.500	0.765	0.909	0.833	0.867	0.846
Threshold: 5.500	0.735	0.955	0.824	0.926	0.859
Threshold: 6.500	0.500	0.977	0.717	0.944	0.769
Threshold: 8.500	0.147	1.000	0.603	1.000	0.628

As results from Tables 5, 6, 7, 8, and 9, a classifier combining the top 8 TSPs achieves a sensitivity of 1 and a specificity of 0.81 in the training set, using a combination threshold of 2.5. This means that a patient is classified at high risk of recurrence when any 2 individual TSPs out of 8 return a high risk prediction. In Figure 2 below are shown the AUC curves for the *k*-TSP classifiers involving the top 8 TSPs:

```
> ####Compute object to plot the ROC/AUC curves for all K-TSP combination
> howManyTSP <- 6:9
> kVec <- paste("k", howManyTSP, sep="")
```

```
> aucGlas <- lapply(k[kVec], plotKTSPauc,
                    data=exprs(glasDataAll.ttm)[, sel], group=glasGroupAll.ttm[sel] )

Call:
roc.default(response = !group, predictor = pred)

Data: pred in 44 controls (!group FALSE) < 34 cases (!group TRUE).
Area under the curve: 0.9485
```

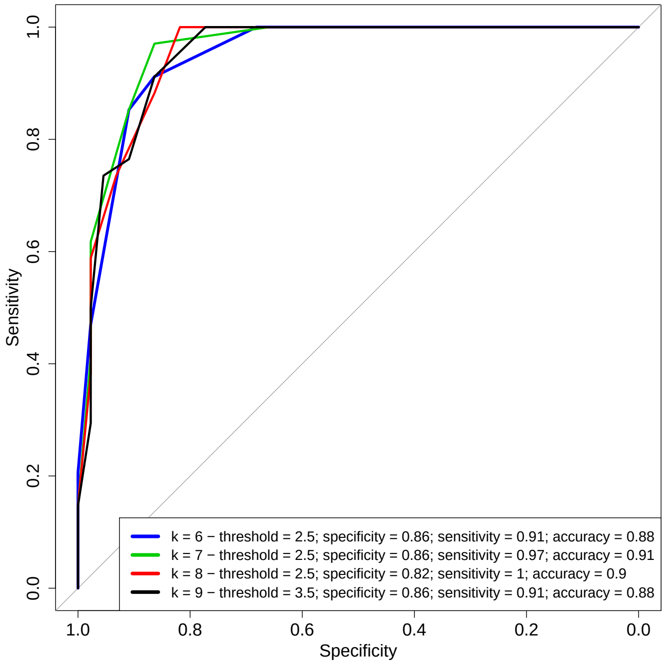

Figure 2: ROC curves for the best k-TSP combinations involving 6 or 7 TSP in the training set.

The following chunk of R was used to train the k-TSP classifier using the van't Veer cases from the Glas cohort, the 70-gene set, and the occurrence of a metastasis within five years as end-point.

```
> ###Trainin the 3-tsp classification within only the 70 genes: complete cohort
> ktspGlasVtV.ttm <- KTSP.Train(exprs(glasDataAll.ttm)[,sel], glasGroupAll.ttm[sel], 8)
```

The individual TSPs constituting the k-TSP classifier are shown below in Table 10.

Table 10: Individual TSP contained in the final classifier.

ALIAS: Bad Gene	ALIAS: Good Gene	COR: Bad Gene	COR: Good Gene	ID: Bad Gene	ID: Good Gene	TSP score
GPR180	GNAZ	-0.38	-0.40	Contig32185_RC	GNAZ	0.60
OXCT1	RTN4RL1	-0.39	0.37	OXCT	Contig46223_RC	0.55
HRASLS	LGP2	-0.36	0.36	LOC57110	FLJ11354	0.53
DTL	RFC4	-0.37	-0.37	L2DTL	RFC4	0.53
MS4A7		0.36	-0.37	CFFM4	Contig40831_RC	0.53
IGFBP5	CDCA7	-0.36	-0.37	IGFBP5	Contig55725_RC	0.52
SERF1A	UCHL5	-0.38	-0.37	SERF1A	UCH37	0.51
MELK	GSTM3	-0.37	0.38	KIAA0175	GSTM3	0.51

The following R code chunk was used to define the k-TSP classifier combination function using the identified optimized 2.5 threshold (see Table 8 above):

```
> ###Combination function
> myCombineFunc <- function(x) sum(x) < 2.5
```

The following R code chunk was used to compute the k-TSP classifier performance in the training set:

```
> ###Create a list to store the results
> ktspResults <- list()
> ###Get resubstitution accuracy: van't Veer cases from the Glas data set
> ktspResults$trainGlasVtV.ttm <- getKTSPperformance(exprs(glasDataAll.ttm)[, sel], ktspGlasVtV.ttm,
                                                      glasGroupAll.ttm[sel], combineFunc = myCombineFunc)
```

Below is shown the k-TSP classifier performance on the training set:

```
> ###Show resubstitution accuracy
> ktspResults$trainGlasVtV.ttm$overallAccuracy

[1] 0.9090909

> ktspResults$trainGlasVtV.ttm

Confusion Matrix and Statistics

   group
     0  1
  0 34  8
  1  0 36

               Accuracy : 0.8974
                 95% CI : (0.8079, 0.9547)
    No Information Rate : 0.5641
    P-Value [Acc > NIR] : 1.403e-10

                  Kappa : 0.7969
 Mcnemar's Test P-Value : 0.01333

            Sensitivity : 1.0000
            Specificity : 0.8182
         Pos Pred Value : 0.8095
         Neg Pred Value : 1.0000
             Prevalence : 0.4359
         Detection Rate : 0.4359
   Detection Prevalence : 0.5385

       'Positive' Class : 0
```

In Figure 3 are shown the prognostic group predictions obtained for each individual patient using each separate TSP from Table 8.

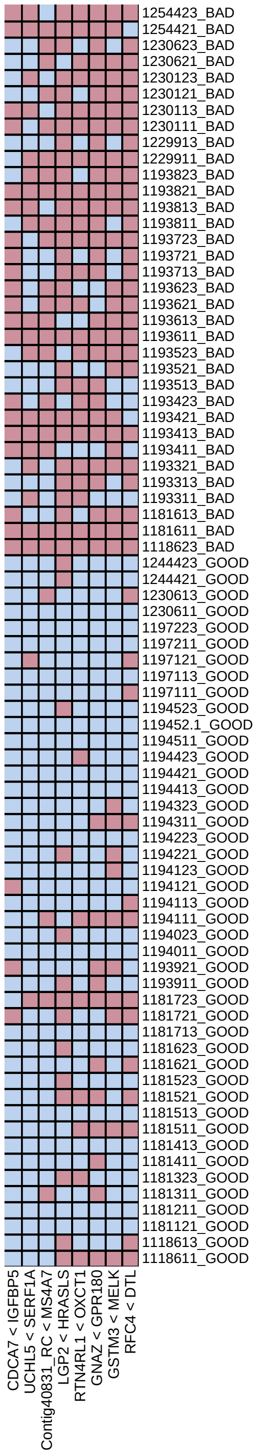

Figure 3: Prognostic group predictions for each separate TSP from Table 8. The TSP are shown as columns, while the distinct patients are represented on the rows. The pink color is used for classification in the "Bad" prognostic group, while blue for "Good" prognostic group predictions.

4.2 Re-training of the MammaPrint classifier

The chunk of R code below shows the steps necessary to re-train the MammaPrint classifier. To this end we need to compute the cosine correlation between each sample and the 70-gene mean expression profile of the 44 patients in the good prognostic group.

```
> ###Load the lsa library, which contains the the cosine correlation between two vectors
> require(lsa)
> ###Obtain mean expression profiles for the 70-gene signature
> gns70profile <- exprs(glasDataAll.ttm)[ , sel]
> gns70profile <- gns70profile[ , glasGroupAll.ttm[sel] == 1]
> gns70profile <- apply(gns70profile, 1, mean, trim=0)
> ###Define the correlation threshold for the final classification
> cosineCorThr <- 0.4
> ###Compute the cosine correlation for each patient
> cosineCorsOnGlas <- apply(exprs(glasDataAll.ttm)[ , sel], 2, cosine, y=gns70profile)
> ###Classify each patient using the specified threshold
> computedMammaPredOnGlas <- 1 * (cosineCorsOnGlas > cosineCorThr)
```

Below is shown the re-substitution performance using the re-trained MammaPrint classifier.

```
> confusionMatrix(computedMammaPredOnGlas, glasGroupAll.ttm[sel])

Confusion Matrix and Statistics

          Reference
Prediction  0  1
         0 30 13
         1  4 31

               Accuracy : 0.7821
                 95% CI : (0.6741, 0.8676)
    No Information Rate : 0.5641
    P-Value [Acc > NIR] : 4.804e-05

                  Kappa : 0.5698
 Mcnemar's Test P-Value : 0.05235

            Sensitivity : 0.8824
            Specificity : 0.7045
         Pos Pred Value : 0.6977
         Neg Pred Value : 0.8857
             Prevalence : 0.4359
         Detection Rate : 0.3846
   Detection Prevalence : 0.5513

       'Positive' Class : 0
```

It must be noted that there is a discrepancy in the predictions obtained with this apporach compared to what was originally reported by Glas and colleagues [3]. In the case of the retrained classifier there are 13 rather than 12 patients with a good prognosis who are misclassified and assigned to the poor prognostic group. Possible explanations for these differencies are:

- The set of patients we have for training purposes might not be exactly the same employed in the original manuscript (for an explanation see Section 3.5);
- The pre-processing strategy we have applied – derived from the description available in the original manuscripts (see Section 3.2.2 for details) – might differ slightly from what performed by Glas and colleagues [3, 14];

- A combination of the two reasons above.

4.3 Development of other classifiers using the Glas data set

For training purposes we have also applied Linear Discriminant Analysis (LDA) and Prediction Analysis of Microarray (PAM) on the Glas data set [3]. Also in this case we have selected and used the same patients and end-point of the original van't Veer study [1].

4.3.1 LDA classifier training

In the `R` code chunk below is shown how to train a prognostic classifier using LDA with the `R-Bioconductor` package `MLInterfaces`. To this end the Glas and Buyse data must be combined in a unique `ExpressionSet` instance, as show below.

```
> ###Load MLInterfaces
> require(MLInterfaces)
> ###Combine Glas and Buyse expression data as required in MLInterfaces
> df1 <- exprs(glasDataAll.ttm[, sel])
> df2 <- exprs(buyseDataAll.ttm)
> glasBuyseData.ttm <- merge(df1, df2, by=0)
> rownames(glasBuyseData.ttm) <- glasBuyseData.ttm[,1]
> glasBuyseData.ttm <- as.matrix(glasBuyseData.ttm[,-1])
> ###Combine Glas and Buyse group information as required in MLInterfaces
> glasBuyseGroup.ttm <- data.frame(group.ttm=factor(c(glasGroupAll.ttm[sel], buyseGroupAll.ttm)))
> levels(glasBuyseGroup.ttm$group.ttm) <- c("Bad", "Good")
> rownames(glasBuyseGroup.ttm) <- colnames(glasBuyseData.ttm)
> ###Create as new ExpressionSet
> glasBuyseData.ttm <- ExpressionSet(glasBuyseData.ttm,
                                     phenoData=AnnotatedDataFrame(glasBuyseGroup.ttm))
> ###Create a numeric index for Glas and Buyse samples in the merged data
> ###as required in MLInterfaces for training and validation purposes
> glassTrainInd <- 1:length(glasGroupAll.ttm[sel])
> ###LDA with MLInterfaces
> lda.res <- MLearn(group.ttm~., glasBuyseData.ttm, .method=ldaI, trainInd=glassTrainInd)
```

Below is shown the LDA classifier performance on the training set:

```
> confusionMatrix(as.table(t(confuMat(lda.res, "train"))))
```

```
Confusion Matrix and Statistics

         given
predicted Bad Good
     Bad   34    0
     Good   0   44

               Accuracy : 1
                 95% CI : (0.9538, 1)
    No Information Rate : 0.5641
    P-Value [Acc > NIR] : < 2.2e-16

                  Kappa : 1
 Mcnemar's Test P-Value : NA

            Sensitivity : 1.0000
            Specificity : 1.0000
```

```
         Pos Pred Value : 1.0000
         Neg Pred Value : 1.0000
             Prevalence : 0.4359
         Detection Rate : 0.4359
   Detection Prevalence : 0.4359

       'Positive' Class : Bad
```

4.3.2 PAM classifier training

In the R code chunk below is shown how to train a prognostic classifier using PAM with the `R-Bioconductor` package `pamr`. Also in this case the data must be assembled to meet the package specifications.

```
> require(pamr)
> ###Prepare the data for PAM analysis
> symbols <- rownames(exprs(glasDataAll.ttm[, sel]))
> pam.glasData <- list(x=exprs(glasDataAll.ttm[, sel]), y=glasGroupAll.ttm[sel],
                      genenames=symbols, geneid=symbols)
> ###Train the PAM classifier
> pam.glasRes <- pamr.train(pam.glasData)
> ###Cross validation
> pam.glasResCV <- pamr.cv(pam.glasRes, pam.glasData)
> ###Predictions in the trainin set using all genes
> pam.glasResPred <- pamr.predict(pam.glasRes, pam.glasData$x, threshold=0)
```

In Figure 4 are shown the shrunken centroids from PAM.

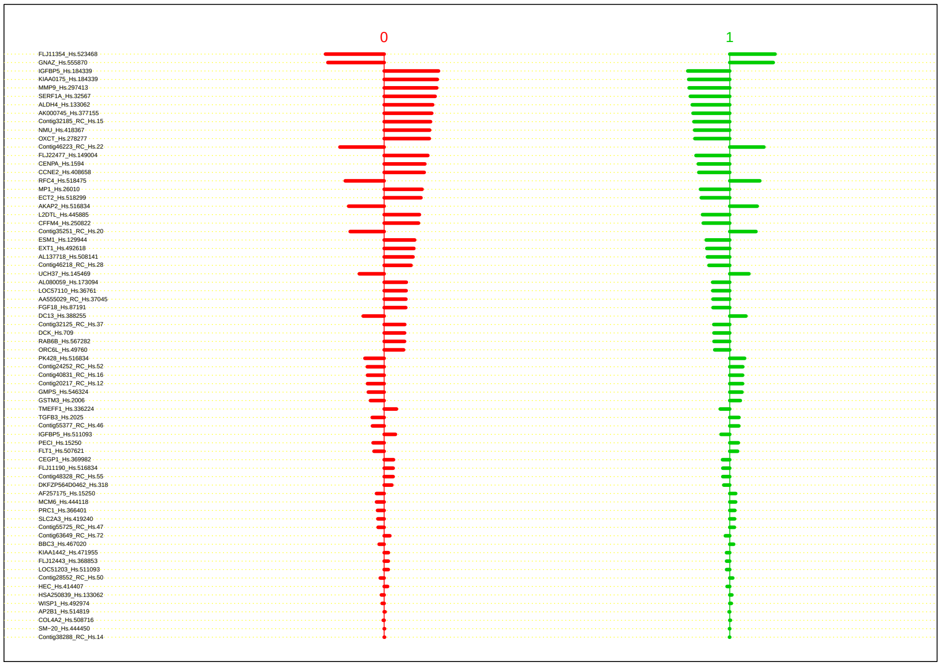

Figure 4: Plot shows the PAM shrunken centroids in the training set (0 = “bad” prognostic group, 1 = “good” prognostic group).

In Figure 5, the plots shows the cross-validation miss-classification error in the training set for decreasing

number of genes in the PAM classifier.

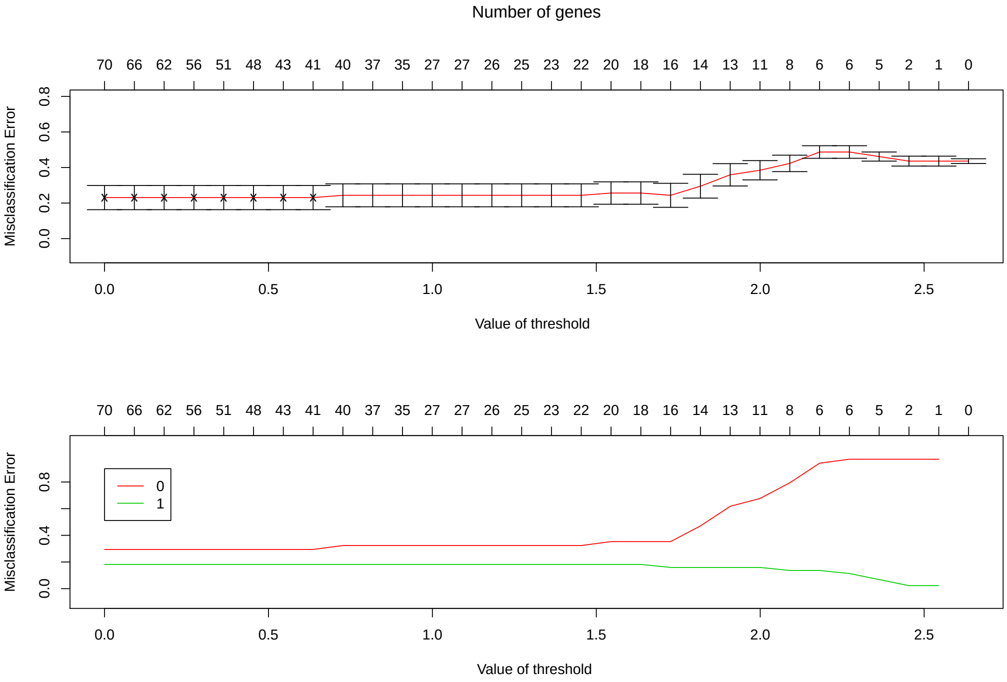

Figure 5: Cross-validation miss-classification error in the training set for decreasing number of genes in the PAM classifier. (0 = “bad” prognostic group, 1 = “good” prognostic group).

Below is shown the PAM classifier performance on the training set:

```
> confusionMatrix(pam.glasResPred, glasGroupAll.ttm[sel])

Confusion Matrix and Statistics

          Reference
Prediction  0  1
         0 24  8
         1 10 36

               Accuracy : 0.7692
                 95% CI : (0.66, 0.8571)
    No Information Rate : 0.5641
    P-Value [Acc > NIR] : 0.0001308

                  Kappa : 0.5276
 Mcnemar's Test P-Value : 0.8136637

            Sensitivity : 0.7059
            Specificity : 0.8182
         Pos Pred Value : 0.7500
         Neg Pred Value : 0.7826
             Prevalence : 0.4359
         Detection Rate : 0.3077
   Detection Prevalence : 0.4103

       'Positive' Class : 0
```

4.4 ROC-AUC analysis in the Glas cohort

The chunk of code below shows how to compute resubstitution estimates in the Glas cohort performing AUC analysis comparing the *k*-TSP classifier, MammaPrint, and the other predictors.

```
> ###Assemble all predictions in a data.frame
> allPredictors <- data.frame(Response=glasGroupAll.ttm[sel],###RESPONSE
                              KTSP=KTSP.Classify(exprs(glasDataAll.ttm)[,sel], #KTSP classifier
                                 ktspGlasVtV.ttm, myCombineFunc), #Threshold maximizing sensitivity
                               retrainedMammaPrint=computedMammaPredOnGlas, #Retrained MammaPrint
                               PAM=-1 + as.numeric(pam.glasResPred), #PAM classifier (subtract 1 for levels consistency
                               LDA=-1 + as.numeric(trainPredictions(lda.res)), #LDA classifier (subtract 1 for levels c
                               stringsAsFactors=FALSE)
> ###In the pROC package the case are usually labeled with 1 and controls with 0
> allPredictors <- lapply(allPredictors, function(x) abs(-1 + as.numeric(x)) )
```

In Figure 6 are shown the resubstitution ROC and AUC results as obtained in the training set for the *k*-TSP predictors.

```
Call:
roc.default(response = resp, predictor = pred)

Data: pred in 44 controls (resp 0) < 34 cases (resp 1).
Area under the curve: 0.9091
```

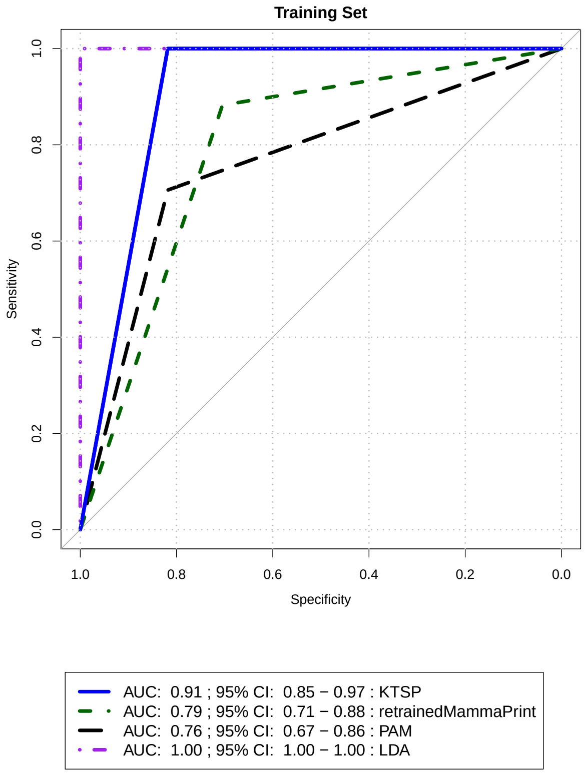

Figure 6: Resubstitution ROC-AUC analysis in the training set of samples (Glas cohort). The blue continuous line depicts the ROC curve for the classification obtained obtained by using the dichotomized *k*-TSP classifier using the 2.5 threshold that maximized sensitivity (see Table 8). AUC along with 95% confidence intervals are shown in the figure legend.

5 Prognostic predictor validation

5.1 *k*-TSP classifier performance in the Buyse cohort

We have validated our *k*-TSP classifier using the Buyse cohort and the same end-point used for training (occurrence of a metastasis within five years as first recurrence event). The following `R` code chunk was used to compute the prognostic prediction accuracy for the selected end-point in this test set, using all 307 cases of the Buyse cohort.

```
> ###Get the validation accuracy in Buyse (all cases)
> ktspResults$testBuyseAll.ktspGlasVtV.ttm <- getKTSPperformance(exprs(buyseEset), ktspGlasVtV.ttm,
                                                                  buyseGroupAll.ttm,
                                                                  combineFunc = myCombineFunc)
```

The prognostic prediction accuracy of the *k*-TSP classifier for the development of metastasis as first recurrence within 5 years for all the 307 cases is shown below:

```
> ###Get the validation test accuracy: TTM by classifier from Glas (all cases)
> ktspResults$testBuyseAll.ktspGlasVtV.ttm$overallAccuracy

[1] 0.6920622

> ktspResults$testBuyseAll.ktspGlasVtV.ttm

Confusion Matrix and Statistics

   group
      0   1
  0  43 138
  1   4 122

               Accuracy : 0.5375
                 95% CI : (0.4799, 0.5943)
    No Information Rate : 0.8469
    P-Value [Acc > NIR] : 1

                  Kappa : 0.1772
 Mcnemar's Test P-Value : <2e-16

            Sensitivity : 0.9149
            Specificity : 0.4692
         Pos Pred Value : 0.2376
         Neg Pred Value : 0.9683
             Prevalence : 0.1531
         Detection Rate : 0.1401
   Detection Prevalence : 0.5896

       'Positive' Class : 0
```

In Figures 7 8 are shown the prognostic group predictions obtained for each individual patient using each separate TSP from Table 8.

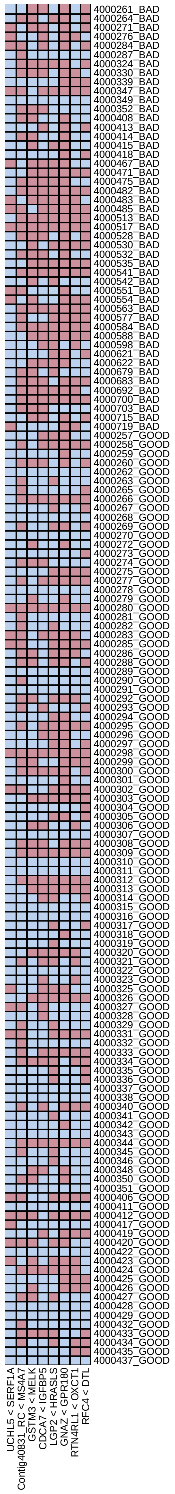

Figure 7: Prognostic group predictions for each separate TSP from Table 8. The first set of 150 patients is shown. The TSP are shown as columns, while the distinct patients are represented on the rows. The pink color is used for classification in the "Bad" prognostic group, while blue for "Good" prognostic group predictions.

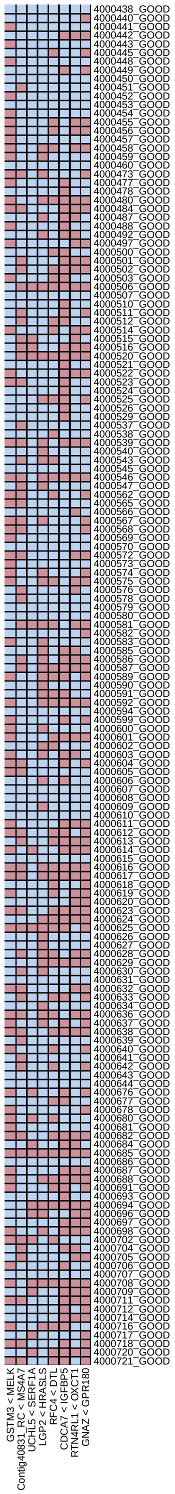

Figure 8: Prognostic group predictions for each separate TSP from Table 8. The second set of 157 patients is shown. The TSP are shown as columns, while the distinct patients are represented on the rows. The pink color is used for classification in the “Bad” prognostic group, while blue for “Good” prognostic group predictions.

5.2 MammaPrint assay performance in the Buyse cohort

5.2.1 Reported MammaPrint predictions

We have also evaluated the performance of the MammaPrint assay using the prediction information provided with the ArrayExpress "E-TABM-77" series for the Buyse cohort. The following R code chunk was used to compute the prediction accuracy for the selected end-point in the Buyse cohort, using all 307 cases.

```
> ###Extract and format the MammaPrint prediction results
> mammaPrintPredictionAll <- pData(buyseEset)$Factor.Value.MammaPrint.prediction
> mammaPrintPredictionAll <- factor(gsub("\\s.+", "", gsub(".+:", "", mammaPrintPredictionAll)))
```

```
> ###Summary of MammaPrint results on Buyse
> table(mammaPrintPredictionAll)
```

```
mammaPrintPredictionAll
high  low
 194  113
```

```
> levels(mammaPrintPredictionAll) <- 0:1
```

The prognostic prediction accuracy of the MammaPrint assay for the development of metastasis as first recurrence within 5 years for the all 307 cases is shown below:

```
> ###Computing validation accuracy: TTM by MammaPrint (all 307 cases)
> mammaPrintValidAll <- confusionMatrix(table(mammaPrintPredictionAll, buyseGroupAll.ttm))
> mean(mammaPrintValidAll$byClass[c("Sensitivity", "Specificity")])
```

```
[1] 0.6545008
```

```
> mammaPrintValidAll
```

```
Confusion Matrix and Statistics

                       buyseGroupAll.ttm
mammaPrintPredictionAll   0   1
                      0  42 152
                      1   5 108

               Accuracy : 0.4886
                 95% CI : (0.4314, 0.546)
    No Information Rate : 0.8469
    P-Value [Acc > NIR] : 1

                  Kappa : 0.1355
 Mcnemar's Test P-Value : <2e-16

            Sensitivity : 0.8936
            Specificity : 0.4154
         Pos Pred Value : 0.2165
         Neg Pred Value : 0.9558
             Prevalence : 0.1531
         Detection Rate : 0.1368
   Detection Prevalence : 0.6319

       'Positive' Class : 0
```

5.2.2 Retrained MammaPrint predictions

The chunk of Rcode below was used to predict the prognostic group using the retrained MammaPrint classifier and the selectect threshold of 0.4.

```
> ###Compute the cosine correlation for each patient
> cosineCorsOnBuyse <- apply(exprs(buyseDataAll.ttm), 2, cosine, y=gns70profile)
> ###Classify each patient using the specified threshold
> computedMammaPredOnBuyse <- 1 * (cosineCorsOnBuyse > cosineCorThr)
```

Below is shown the performance using the re-trained MammaPrint classifier in the Buyse cohort.

```
> confusionMatrix(computedMammaPredOnBuyse, buyseGroupAll.ttm)

Confusion Matrix and Statistics

          Reference
Prediction   0   1
         0  44 176
         1   3  84

               Accuracy : 0.4169
                 95% CI : (0.3612, 0.4743)
    No Information Rate : 0.8469
    P-Value [Acc > NIR] : 1

                  Kappa : 0.1034
 Mcnemar's Test P-Value : <2e-16

            Sensitivity : 0.9362
            Specificity : 0.3231
         Pos Pred Value : 0.2000
         Neg Pred Value : 0.9655
             Prevalence : 0.1531
         Detection Rate : 0.1433
   Detection Prevalence : 0.7166

       'Positive' Class : 0
```

The chunk of code below is used to show the disagreement between the predictions recomputed using the retrained MammaPrint signature and the prediction reported in ArrayExpress "E-TABM-77" record (Buyse cohort). As previously noted about the performance of the retrained classifier in the Glas cohort, also in this case there are several discrepancies between the predictions reported for the MammaPrint assay and those obtained with the retrained classifier. As mentioned above, possible explanations for such differencies are likely due to differencies in clinical sample annotation, or in gene expression data preprocessing (see 4.2).

```
> ###Evaluate classification on the Buyse cohort
> reportedMamma <- pData(buyseDataAll.ttm)$Factor.Value.MammaPrint.prediction
> table(factor(reportedMamma))

MammaPrint result:high risk  MammaPrint result:low risk
                        194                         113

> reportedMamma <- as.numeric(factor(reportedMamma)) - 1
> table(factor(reportedMamma))

  0   1
194 113

> table(Computed=computedMammaPredOnBuyse,
       Reported=reportedMamma)
```

```
         Reported
Computed   0   1
       0 185  35
       1   9  78
```

5.3 Performance of other classifiers in the Buyse cohort

5.3.1 LDA performance in the Buyse cohort

Below is shown the performance using the LDA classifier in the Buyse cohort. The poor performance of LDA classifier in the validation cohort is likely due to over-fitting due to the large number of parameters that must be estimated.

```
> confusionMatrix(as.table(t(confuMat(lda.res))))

Confusion Matrix and Statistics

         given
predicted Bad Good
     Bad   33  193
     Good  14   67

               Accuracy : 0.3257
                 95% CI : (0.2736, 0.3813)
    No Information Rate : 0.8469
    P-Value [Acc > NIR] : 1

                  Kappa : -0.0157
 Mcnemar's Test P-Value : <2e-16

            Sensitivity : 0.7021
            Specificity : 0.2577
         Pos Pred Value : 0.1460
         Neg Pred Value : 0.8272
             Prevalence : 0.1531
         Detection Rate : 0.1075
   Detection Prevalence : 0.7362

       'Positive' Class : Bad
```

5.3.2 PAM performance in the Buyse cohort

Below is shown the performance using the PAM classifier in the Buyse cohort.

```
> pam.buyseResPred <- pamr.predict(pam.glasRes, newx=exprs(buyseDataAll.ttm),
                        threshold=0, type="class")
> confusionMatrix(pam.buyseResPred, buyseGroupAll.ttm )

Confusion Matrix and Statistics

          Reference
Prediction   0   1
         0  39 107
         1   8 153

               Accuracy : 0.6254
```

```
                 95% CI : (0.5686, 0.6797)
    No Information Rate : 0.8469
    P-Value [Acc > NIR] : 1

                  Kappa : 0.2245
 Mcnemar's Test P-Value : <2e-16

            Sensitivity : 0.8298
            Specificity : 0.5885
         Pos Pred Value : 0.2671
         Neg Pred Value : 0.9503
             Prevalence : 0.1531
         Detection Rate : 0.1270
   Detection Prevalence : 0.4756

       'Positive' Class : 0
```

5.4 Performance of clinical classifiers in the Buyse cohort

5.4.1 Adjuvan! online performance in the Buyse cohort

Below is shown the performance of the Adjuvan! online classifier in the Buyse cohort.

```
> ###Adjuvant online risk groups in the Buyse cohort
> Adjuvant <- factor(pData(buyseDataAll.ttm)$Factor.Value.Adjuvant.online.risk.status,
                  levels=c("Adjuvant online:high clinical risk", "Adjuvant online:low clinical risk"))
> levels(Adjuvant) <- 0:1
> confusionMatrix(Adjuvant, buyseGroupAll.ttm )
```

```
Confusion Matrix and Statistics

          Reference
Prediction   0   1
         0  41 181
         1   6  79

               Accuracy : 0.3909
                 95% CI : (0.336, 0.4479)
    No Information Rate : 0.8469
    P-Value [Acc > NIR] : 1

                  Kappa : 0.0698
 Mcnemar's Test P-Value : <2e-16

            Sensitivity : 0.8723
            Specificity : 0.3038
         Pos Pred Value : 0.1847
         Neg Pred Value : 0.9294
             Prevalence : 0.1531
         Detection Rate : 0.1336
   Detection Prevalence : 0.7231

       'Positive' Class : 0
```

5.4.2 NPI performance in the Buyse cohort

Below is shown the performance of the Nottingham Prognostic Index (NPI) in the Buyse cohort.

```
> ###NPI risk groups in the Buyse cohort
> NPIrisk <- factor(pData(buyseDataAll.ttm)$Factor.Value.NPI.Risk.Status,
                   levels=c("NPI:high clinical risk", "NPI:low clinical risk", "NPI:not applicable"))
> levels(NPIrisk) <- c(0, 1, NA)
> confusionMatrix(NPIrisk, buyseGroupAll.ttm )
```

```
Confusion Matrix and Statistics

          Reference
Prediction   0   1
         0  37 132
         1   9 121

               Accuracy : 0.5284
                 95% CI : (0.4701, 0.5862)
    No Information Rate : 0.8462
    P-Value [Acc > NIR] : 1

                  Kappa : 0.135
 Mcnemar's Test P-Value : <2e-16

            Sensitivity : 0.8043
            Specificity : 0.4783
         Pos Pred Value : 0.2189
         Neg Pred Value : 0.9308
             Prevalence : 0.1538
         Detection Rate : 0.1237
   Detection Prevalence : 0.5652

       'Positive' Class : 0
```

5.4.3 St Gallen performance in the Buyse cohort

Below is shown the performance of the St. Gallen criteria in the Buyse cohort.

```
> ###St.Gallen risk groups in the Buyse cohort
> StGallen <- factor(pData(buyseDataAll.ttm)$Factor.Value.St.Gallen.Risk.Status,
                    levels=c("St Gallen category:high clinical risk", "St Gallen category:low clinical risk",
                    "St Gallen category:not applicable"))
> levels(StGallen) <- c(0, 1, NA)
> confusionMatrix(StGallen, buyseGroupAll.ttm )
```

```
Confusion Matrix and Statistics

          Reference
Prediction   0   1
         0  46 228
         1   1  26

               Accuracy : 0.2392
                 95% CI : (0.1921, 0.2915)
    No Information Rate : 0.8439
    P-Value [Acc > NIR] : 1

                  Kappa : 0.0273
```

```
Mcnemar's Test P-Value : <2e-16

            Sensitivity : 0.9787
            Specificity : 0.1024
         Pos Pred Value : 0.1679
         Neg Pred Value : 0.9630
             Prevalence : 0.1561
         Detection Rate : 0.1528
   Detection Prevalence : 0.9103

       'Positive' Class : 0
```

5.5 ROC-AUC analysis in the Buyse cohort

The chunk of code below shows how to compute resubstitution estimates in the Buyse cohort performing AUC analysis comparing the k-TSP classifier, MammaPrint, the other molecular predictors, and the clinical predictors (Adjuvant! online, Nottingham Prognostic Index and the St Gallen criteria).

```
> ###Assemble all predictions in a data.frame
> allPredictorsTest <- data.frame(Response=buyseGroupAll.ttm,
                                  KTSP=KTSP.Classify(exprs(buyseDataAll.ttm), #KTSP classifier
                                     ktspGlasVtV.ttm, myCombineFunc), #Threshold maximizing sensitivity
                                  retrainedMammaPrint=computedMammaPredOnBuyse, #Retrained MammaPrint
                                  reportedMammaPrintBuyse=reportedMamma, #MammaPrint as reported
                                  PAM=-1 + as.numeric(pam.buyseResPred), #PAM classifier (subtract 1 for levels consist
                                  LDA=-1 + as.numeric(testPredictions(lda.res)), #LDA classifier (subtract 1 for levels
                                  NPIrisk=-1 + as.numeric(NPIrisk), #NPI (subtract 1 for levels consistency)
                                  StGallen=-1 + as.numeric(StGallen), #StGallen classifier (subtract 1 for levels consi
                                  Adjuvant=-1 + as.numeric(Adjuvant), #Adjuvant classifier (subtract 1 for levels consi
                                  stringsAsFactors=FALSE)
> ###In the pROC package the case are usually labeled with 1 and controls with 0
> allPredictorsTest <- lapply(allPredictorsTest, function(x) abs(-1 + as.numeric(x)) )
```

In Figure 9 are shown results from the ROC and AUC analysis as obtained in the test set for the k-TSP predictors, MammaPrint, the other molecular predictors, and the clinical based classifiers (Adjuvant online, NPI and the St. Gallen criteria).

```
Call:
roc.default(response = resp, predictor = pred)

Data: pred in 260 controls (resp 0) < 47 cases (resp 1).
Area under the curve: 0.6921
```

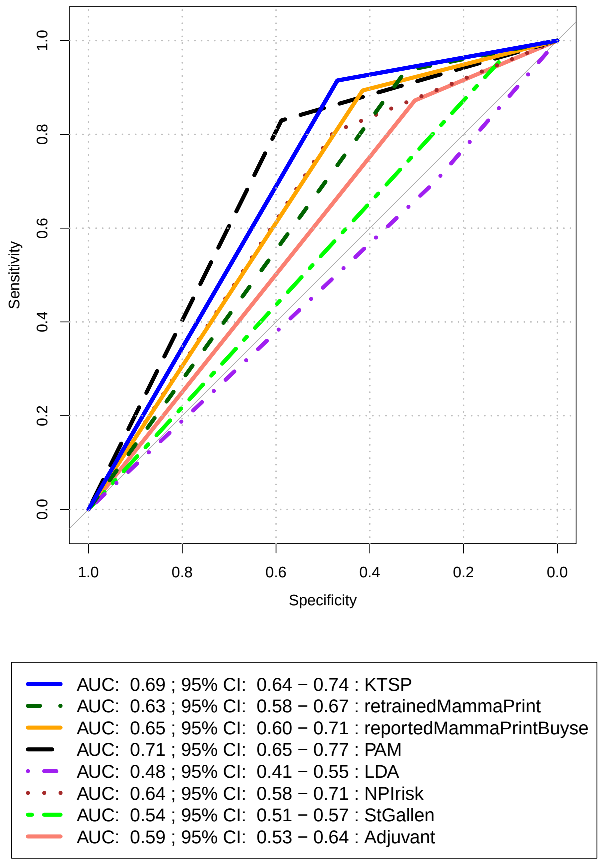

Figure 9: ROC-AUC analysis in the test set of samples (Buyse cohort). The blue continuous line depicts the ROC curve for the classification obtained obtained by using the dichotomized k-TSP classifier using the 2.5 threshold that maximized sensitivity (see Table 8). The legend list the colors for all other lines and the AUC values for all the predictors, along with 95% confidence intervals, are shown in the figure legend.

5.6 Survival analysis in the Buyse cohorts

5.6.1 Time to development of metastasis

Prepare the data for Kaplan-Meier analysis stratified by MammaPrint and K-TSP prediction results for time to development of metastasis as first recurrence event.

```
> ###Require survival library
> require(survival)
> ###Extract Buyse phenotypic information
> pBuyse <- pData(buyseDataAll.ttm)
> ###Assemble Survival object for time to metastasis
> survPH.ttm <- Surv(pBuyse$TTM , pBuyse$TTMevent)
```

The R code chunk below was used to compute the Kaplan-Meier curves for time to metastasis development stratified by the KTSP prediction.

```
> ###Obtain and fromat KTSP prediction: set high-risk equal to 1
> ktspPrediction <- factor(1 * ! KTSP.Classify(exprs(buyseDataAll.ttm), ktspGlasVtV.ttm, combineFunc=myCombineFunc))
> levels(ktspPrediction) <- c("Low", "High")
> ###Kaplan-Meier curves stratified by KTSP
> ktspKM.ttm <- survfit(survPH.ttm~strata(ktspPrediction))
```

The R code chunk below was used to compute univariate Cox proportiona hazard model for time to metastasis development stratified by the KTSP prediction.

```
> ###Cox proportional hazard model with KTSP
> ktspCOX.ttm <- coxph(survPH.ttm ~ ktspPrediction)
```

Below the results of the univariate Cox proportiona hazard model are shown:

```
> ###Cox proportional hazard model results
> summary(ktspCOX.ttm)

Call:
coxph(formula = survPH.ttm ~ ktspPrediction)

  n= 307, number of events= 77

                     coef exp(coef) se(coef)     z Pr(>|z|)
ktspPredictionHigh 0.5408    1.7174   0.2437 2.219   0.0265 *
---
Signif. codes:  0 '***' 0.001 '**' 0.01 '*' 0.05 '.' 0.1 ' ' 1

                   exp(coef) exp(-coef) lower .95 upper .95
ktspPredictionHigh     1.717     0.5823     1.065     2.769

Concordance= 0.598  (se = 0.03 )
Rsquare= 0.017   (max possible= 0.932 )
Likelihood ratio test= 5.19  on 1 df,   p=0.02271
Wald test            = 4.93  on 1 df,   p=0.02647
Score (logrank) test = 5.05  on 1 df,   p=0.02469
```

In Figure 10 are shown Kaplan-Meier curves for time to metastasis development stratified by the KTSP prediction.

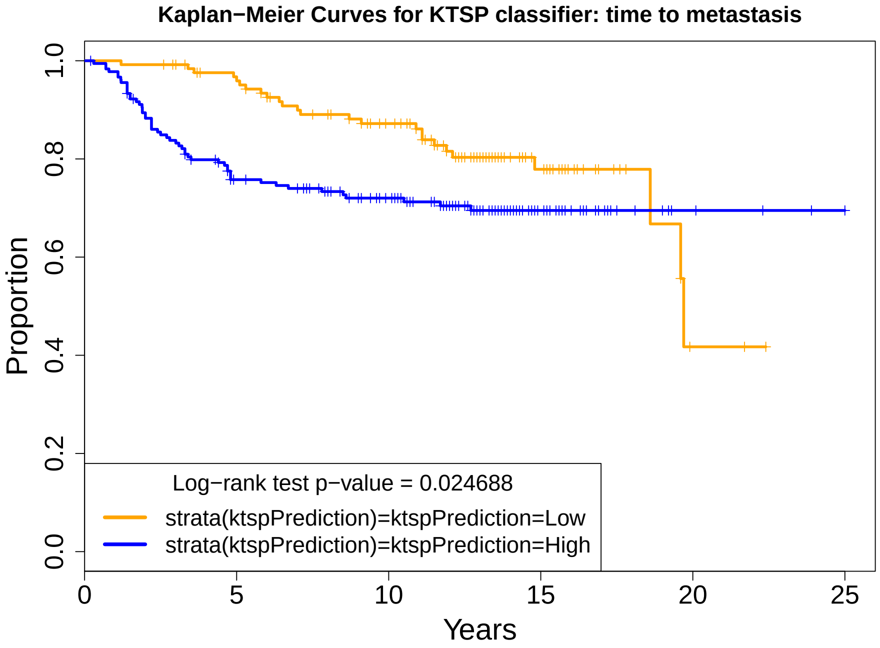

Figure 10: Kaplan-Meier curves for k-TSP prediction: time to development of metastasis.

The R code chunk below was used to compute Kaplan-Meier curves for time to metastasis development as stratified by the MammaPrint prediction.

```
> ###Format MammaPrint prediction: set Low-risk as the first levels
> mammaPrintPredictionAll <- factor(mammaPrintPredictionAll, levels=1:0)
> levels(mammaPrintPredictionAll) <- c("Low", "High")
> ###Kaplan-Meier curves stratified by MammaPrint
> mammaPrintKM.ttm <- survfit(survPH.ttm~strata(mammaPrintPredictionAll))
```

The R code chunk below was used to sompute univariate Cox proportiona hazard model for time to metastasis development stratified by the MammaPrint prediction.

```
> ###Cox proportional hazard model with MammaPrint
> mammaPrintCOX.ttm <- coxph(survPH.ttm ~ mammaPrintPredictionAll)
```

Below the results of the univariate Cox proportional hazard model are shown:

```
> ###Cox proportional hazard model results
> summary(mammaPrintCOX.ttm)
```

```
Call:
coxph(formula = survPH.ttm ~ mammaPrintPredictionAll)

  n= 307, number of events= 77

                              coef exp(coef) se(coef)     z Pr(>|z|)
mammaPrintPredictionAllHigh 0.8173    2.2644   0.2696 3.032  0.00243 **
---
Signif. codes:  0 '***' 0.001 '**' 0.01 '*' 0.05 '.' 0.1 ' ' 1

                            exp(coef) exp(-coef) lower .95 upper .95
mammaPrintPredictionAllHigh     2.264     0.4416     1.335     3.841
```

```
Concordance= 0.613  (se = 0.029 )
Rsquare= 0.033   (max possible= 0.932 )
Likelihood ratio test= 10.42  on 1 df,   p=0.001244
Wald test            = 9.19  on 1 df,   p=0.002433
Score (logrank) test = 9.71  on 1 df,   p=0.001833
```

In Figure 11 Kaplan-Meier curves for time to metastasis stratified by the MammaPrint prediction.

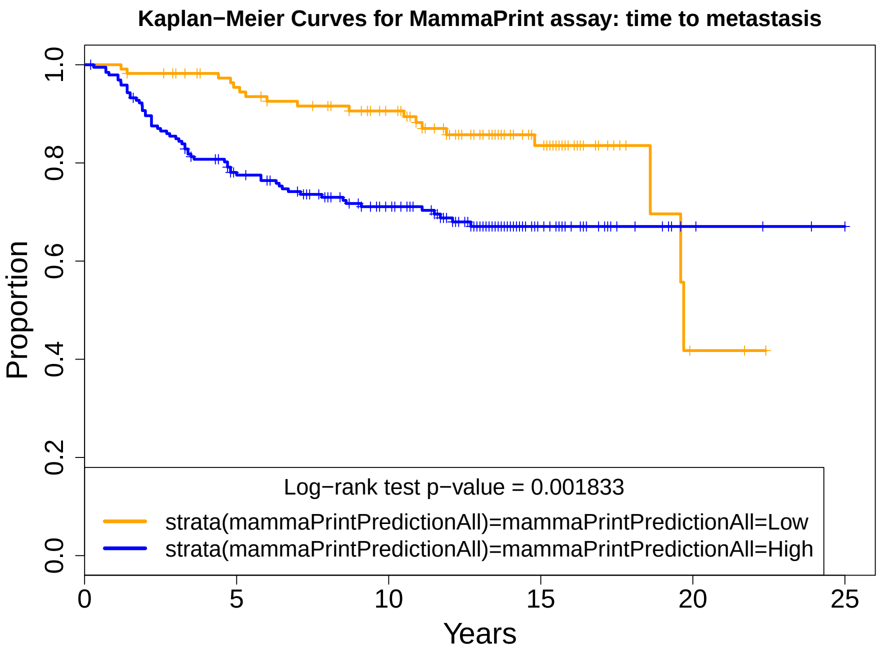

Figure 11: Kaplan-Meier curves for the mammaPrint prediction: time to development of metastasis.

The R code chunk below was used to compute Kaplan-Meier curves for time to metastasis development as stratified by the LDA classifier.

```
> ###Format pam prediction: set Low-risk as the first levels
> ldaTestPred <- testPredictions(lda.res)
> ldaPredictionAll <- factor(ldaTestPred, levels=c("Good", "Bad"))
> levels(ldaPredictionAll) <- c("Low", "High")
> ###Kaplan-Meier curves stratified by PAM
> ldaKM.ttm <- survfit(survPH.ttm~strata(ldaPredictionAll))
```

The R code chunk below was used to sompute univariate Cox proportiona hazard model for time to metastasis development stratified by the LDA prediction.

```
> ###Cox proportional hazard model with LDA
> ldaCOX.ttm <- coxph(survPH.ttm ~ ldaPredictionAll)
```

Below the results of the univariate Cox proportional hazard model are shown:

```
> ###Cox proportional hazard model results
> summary(ldaCOX.ttm)

Call:
coxph(formula = survPH.ttm ~ ldaPredictionAll)


  n= 307, number of events= 77
```

```
                         coef exp(coef) se(coef)      z Pr(>|z|)
ldaPredictionAllHigh -0.07594   0.92687  0.26090 -0.291    0.771

                     exp(coef) exp(-coef) lower .95 upper .95
ldaPredictionAllHigh    0.9269      1.079    0.5558     1.546

Concordance= 0.509  (se = 0.026 )
Rsquare= 0   (max possible= 0.932 )
Likelihood ratio test= 0.08  on 1 df,   p=0.7723
Wald test            = 0.08  on 1 df,   p=0.771
Score (logrank) test = 0.08  on 1 df,   p=0.7709
```

In Figure 12 Kaplan-Meier curves for time to metastasis stratified by the LDA prediction.

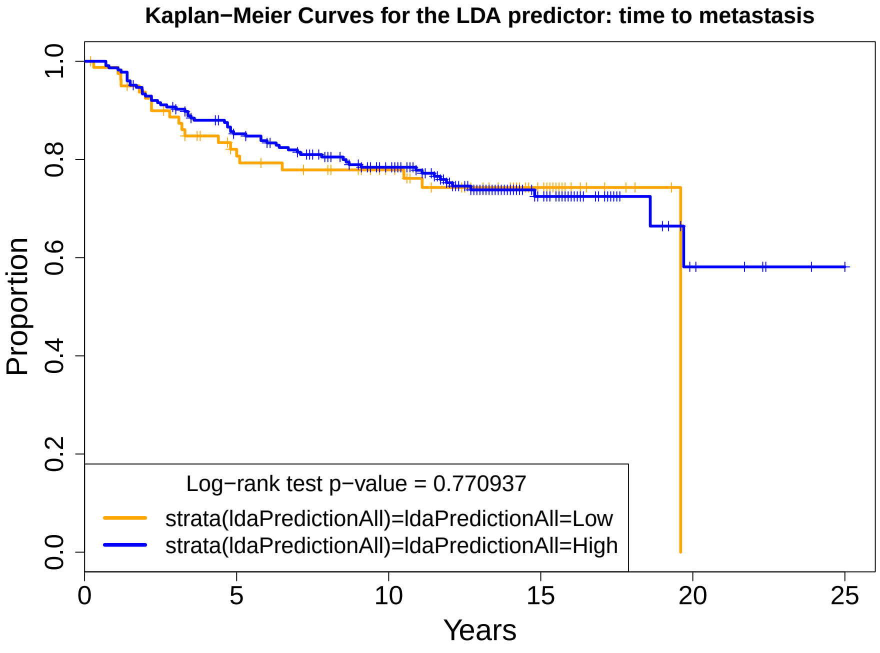

Figure 12: Kaplan-Meier curves for the LDA prediction: time to development of metastasis.

The R code chunk below was used to compute Kaplan-Meier curves for time to metastasis development as stratified by the PAM classifier.

```
> ###Format pam prediction: set Low-risk as the first levels
> pamPredictionAll <- factor(pam.buyseResPred, levels=1:0)
> levels(pamPredictionAll) <- c("Low", "High")
> ###Kaplan-Meier curves stratified by PAM
> pamKM.ttm <- survfit(survPH.ttm~strata(pamPredictionAll))
```

The R code chunk below was used to sompute univariate Cox proportiona hazard model for time to metastasis development stratified by the PAM prediction.

```
> ###Cox proportional hazard model with PAM
> pamCOX.ttm <- coxph(survPH.ttm ~ pamPredictionAll)
```

Below the results of the univariate Cox proportional hazard model are shown:

```
> ###Cox proportional hazard model results
> summary(pamCOX.ttm)

Call:
coxph(formula = survPH.ttm ~ pamPredictionAll)

  n= 307, number of events= 77

                        coef exp(coef) se(coef)     z Pr(>|z|)
pamPredictionAllHigh 0.6269    1.8717   0.2315 2.707  0.00678 **
---
Signif. codes:  0 '***' 0.001 '**' 0.01 '*' 0.05 '.' 0.1 ' ' 1

                     exp(coef) exp(-coef) lower .95 upper .95
pamPredictionAllHigh     1.872     0.5343     1.189     2.947

Concordance= 0.612  (se = 0.029 )
Rsquare= 0.024   (max possible= 0.932 )
Likelihood ratio test= 7.47  on 1 df,   p=0.006268
Wald test            = 7.33  on 1 df,   p=0.00678
Score (logrank) test = 7.57  on 1 df,   p=0.00593
```

In Figure 13 Kaplan-Meier curves for time to metastasis stratified by the PAM prediction.

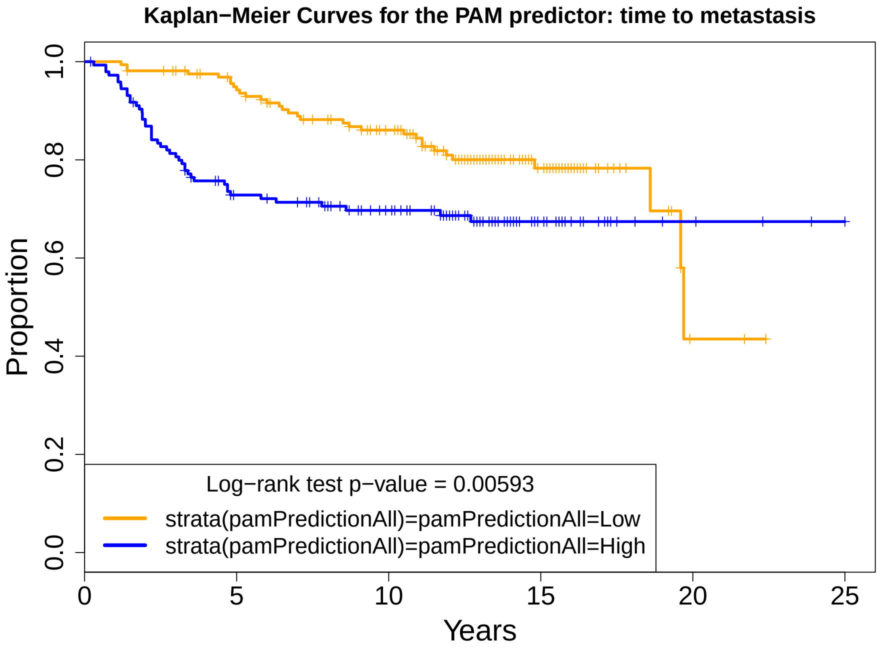

Figure 13: Kaplan-Meier curves for the PAM prediction: time to development of metastasis.

5.6.2 Disease free survival

Prepare the data for Kaplan-Meier analysis stratified by MammaPrint and K-TSP prediction results for disease free survival.

```
> ###Disease free survival
> survPH.dfs <- Surv(pBuyse$DFS , pBuyse$DFSevent)
```

The R code chunk below was used to compute the Kaplan-Meier curves for disease free survival as stratified by the KTSP prediction.

```
> ###Kaplan-Meier curves stratified by KTSP
> ktspKM.dfs <- survfit(survPH.dfs~strata(ktspPrediction))
```

The R code chunk below was used to compute the univariate Cox proportional hazard model for the disease free survival time as stratified by the KTSP prediction.

```
> ###Cox proportional hazard model with KTSP
> ktspCOX.dfs <- coxph(survPH.dfs ~ ktspPrediction)
```

Below the results of the univariate Cox proportional hazard model are shown:

```
> ###Cox proportional hazard model results
> summary(ktspCOX.dfs)

Call:
coxph(formula = survPH.dfs ~ ktspPrediction)

  n= 307, number of events= 139

                     coef exp(coef) se(coef)     z Pr(>|z|)
ktspPredictionHigh 0.3578    1.4302   0.1761 2.032   0.0421 *
---
Signif. codes:  0 '***' 0.001 '**' 0.01 '*' 0.05 '.' 0.1 ' ' 1

                   exp(coef) exp(-coef) lower .95 upper .95
ktspPredictionHigh      1.43     0.6992     1.013      2.02

Concordance= 0.561  (se = 0.022 )
Rsquare= 0.014   (max possible= 0.992 )
Likelihood ratio test= 4.24  on 1 df,   p=0.03943
Wald test            = 4.13  on 1 df,   p=0.04213
Score (logrank) test = 4.17  on 1 df,   p=0.04104
```

In Figure 14 are shown Kaplan-Meier curves for disease free survival ad stratified by the KTSP prediction.

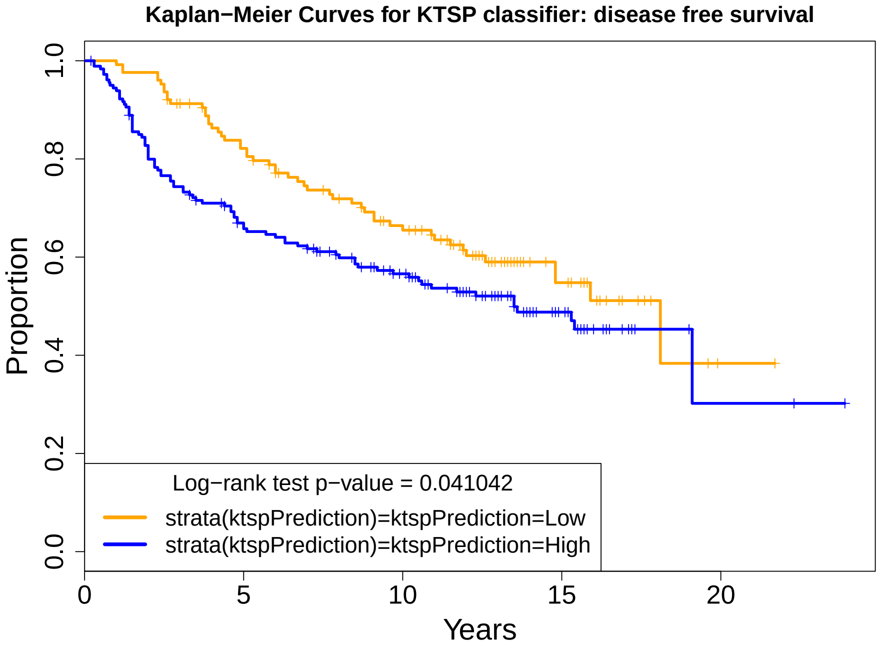

Figure 14: Kaplan-Meier curves for k-TSP prediction: disease free survival.

The R code chunk below was used to compute the Kaplan-Meier curves for disease free survival as stratified by the MammaPrint prediction.

```
> ###Kaplan-Meier curves stratified by MammaPrint
> mammaPrintKM.dfs <- survfit(survPH.dfs~strata(mammaPrintPredictionAll))
```

The R code chunk below was used to compute the univariate Cox proportional hazard model for disease free survival as stratified by the MammaPrint prediction.

```
> ###Cox proportional hazard model with KTSP
> mammaPrintCOX.dfs <- coxph(survPH.dfs ~ mammaPrintPredictionAll)
```

Below the results of the univariate Cox proportional hazard model are shown:

```
> ###Cox proportional hazard model results
> summary(mammaPrintCOX.dfs)

Call:
coxph(formula = survPH.dfs ~ mammaPrintPredictionAll)

  n= 307, number of events= 139

                              coef exp(coef) se(coef)     z Pr(>|z|)
mammaPrintPredictionAllHigh 0.4101    1.5070   0.1826 2.246   0.0247 *
---
Signif. codes:  0 '***' 0.001 '**' 0.01 '*' 0.05 '.' 0.1 ' ' 1

                            exp(coef) exp(-coef) lower .95 upper .95
mammaPrintPredictionAllHigh     1.507     0.6636     1.054     2.155

Concordance= 0.564  (se = 0.022 )
Rsquare= 0.017   (max possible= 0.992 )
Likelihood ratio test= 5.27  on 1 df,   p=0.02168
```

```
Wald test            = 5.04  on 1 df,   p=0.02471
Score (logrank) test = 5.11  on 1 df,   p=0.02373
```

In Figure 15 Kaplan-Meier curves for disease free survival as stratified by the MammaPrint prediction.

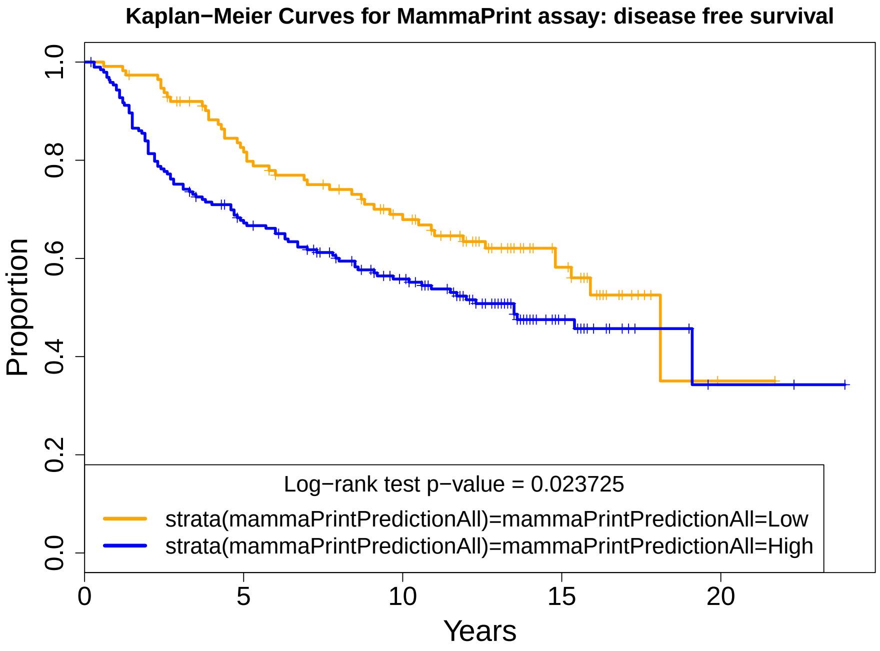

Figure 15: Kaplan-Meier curves for mammaPrint prediction: disease free survival.

The R code chunk below was used to compute the Kaplan-Meier curves for disease free survival as stratified by the LDA prediction.

```
> ###Kaplan-Meier curves stratified by LDA
> ldaKM.dfs <- survfit(survPH.dfs~strata(ldaPredictionAll))
```

The R code chunk below was used to compute the univariate Cox proportional hazard model for disease free survival as stratified by the LDA prediction.

```
> ###Cox proportional hazard model with KTSP
> ldaCOX.dfs <- coxph(survPH.dfs ~ ldaPredictionAll)
```

Below the results of the univariate Cox proportional hazard model are shown:

```
> ###Cox proportional hazard model results
> summary(ldaCOX.dfs)

Call:
coxph(formula = survPH.dfs ~ ldaPredictionAll)

  n= 307, number of events= 139

                        coef exp(coef) se(coef)      z Pr(>|z|)
ldaPredictionAllHigh -0.1168    0.8898   0.1926 -0.606    0.544

                     exp(coef) exp(-coef) lower .95 upper .95
ldaPredictionAllHigh    0.8898      1.124      0.61     1.298
```

```
Concordance= 0.516  (se = 0.019 )
Rsquare= 0.001   (max possible= 0.992 )
Likelihood ratio test= 0.36  on 1 df,   p=0.5481
Wald test            = 0.37  on 1 df,   p=0.5444
Score (logrank) test = 0.37  on 1 df,   p=0.5442
```

In Figure 16 Kaplan-Meier curves for disease free survival as stratified by the LDA prediction.

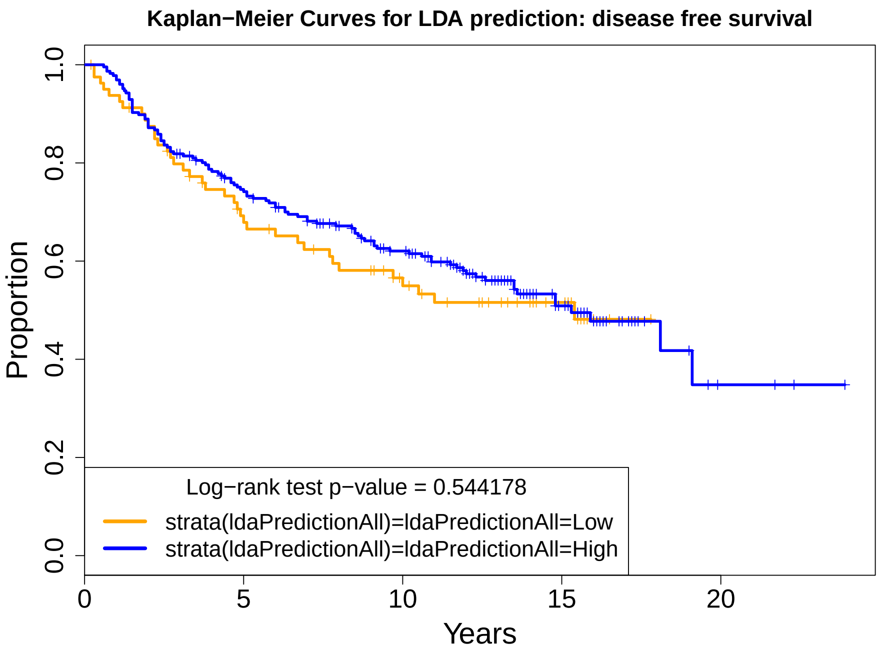

Figure 16: Kaplan-Meier curves for LDAprediction: disease free survival.

The R code chunk below was used to compute the Kaplan-Meier curves for disease free survival as stratified by the PAM prediction.

```
> ###Kaplan-Meier curves stratified by PAM
> pamKM.dfs <- survfit(survPH.dfs~strata(pamPredictionAll))
```

The R code chunk below was used to compute the univariate Cox proportional hazard model for disease free survival as stratified by the PAM prediction.

```
> ###Cox proportional hazard model with KTSP
> pamCOX.dfs <- coxph(survPH.dfs ~ pamPredictionAll)
```

Below the results of the univariate Cox proportional hazard model are shown:

```
> ###Cox proportional hazard model results
> summary(pamCOX.dfs)

Call:
coxph(formula = survPH.dfs ~ pamPredictionAll)

  n= 307, number of events= 139

                       coef exp(coef) se(coef)     z Pr(>|z|)
pamPredictionAllHigh 0.2953    1.3436   0.1698 1.739    0.082 .
---
```

```
Signif. codes:  0 '***' 0.001 '**' 0.01 '*' 0.05 '.' 0.1 ' ' 1

                     exp(coef) exp(-coef) lower .95 upper .95
pamPredictionAllHigh     1.344     0.7443    0.9632     1.874

Concordance= 0.567  (se = 0.022 )
Rsquare= 0.01   (max possible= 0.992 )
Likelihood ratio test= 3.01  on 1 df,   p=0.0827
Wald test            = 3.02  on 1 df,   p=0.08204
Score (logrank) test = 3.05  on 1 df,   p=0.08094
```

In Figure 17 Kaplan-Meier curves for disease free survival as stratified by the PAM prediction.

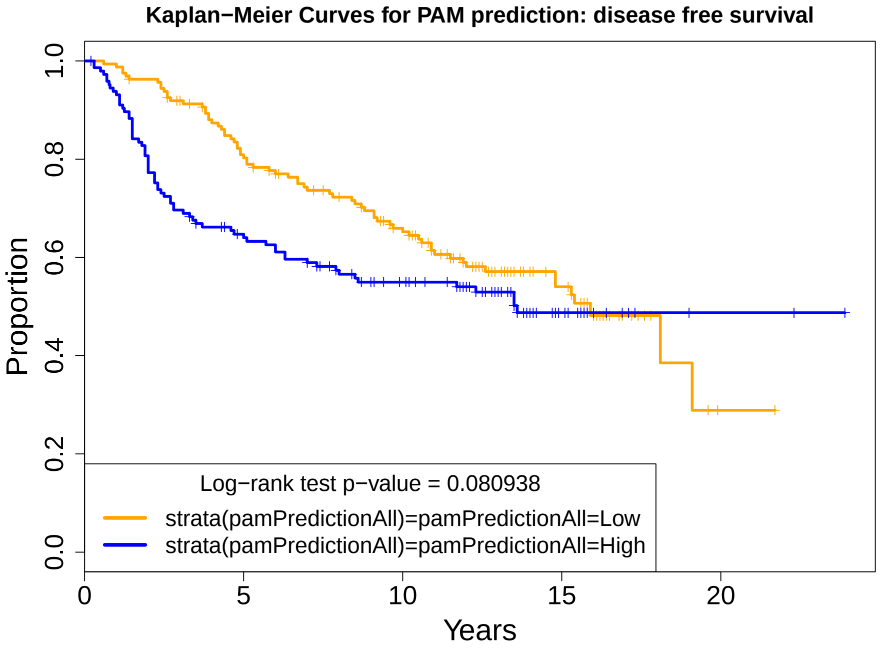

Figure 17: Kaplan-Meier curves for PAMprediction: disease free survival.

5.6.3 Overall survival

Prepare the data for Kaplan-Meier analysis stratified by MammaPrint and K-TSP prediction results for overall survival.

```
> ###Overall survival
> survPH.os <- Surv(pBuyse$OS , pBuyse$OSevent)
```

The R code chunk below was used to compute the Kaplan-Meier curves for overall survival as stratified by the KTSP prediction. x

```
> ###Kaplan-Meier curves stratified by KTSP
> ktspKM.os <- survfit(survPH.os~strata(ktspPrediction))
```

The R code chunk below was used to compute the univariate Cox proportional hazard model for overall survival as stratified by the KTSP prediction.

```
> ###Cox proportional hazard model with KTSP
> ktspCOX.os <- coxph(survPH.os ~ ktspPrediction)
```

Below the results of the univariate Cox proportional hazard model are shown:

```
> ###Cox proportional hazard model results
> summary(ktspCOX.os)

Call:
coxph(formula = survPH.os ~ ktspPrediction)

  n= 307, number of events= 82

                     coef exp(coef) se(coef)     z Pr(>|z|)
ktspPredictionHigh 0.6665    1.9473   0.2430 2.743  0.00609 **
---
Signif. codes:  0 '***' 0.001 '**' 0.01 '*' 0.05 '.' 0.1 ' ' 1

                   exp(coef) exp(-coef) lower .95 upper .95
ktspPredictionHigh     1.947     0.5135      1.21     3.135

Concordance= 0.607  (se = 0.029 )
Rsquare= 0.026   (max possible= 0.941 )
Likelihood ratio test= 8.12  on 1 df,   p=0.00437
Wald test            = 7.52  on 1 df,   p=0.006092
Score (logrank) test = 7.8  on 1 df,   p=0.005215
```

In Figure 18 are shown Kaplan-Meier curves for overall survival as stratified by the KTSP prediction.

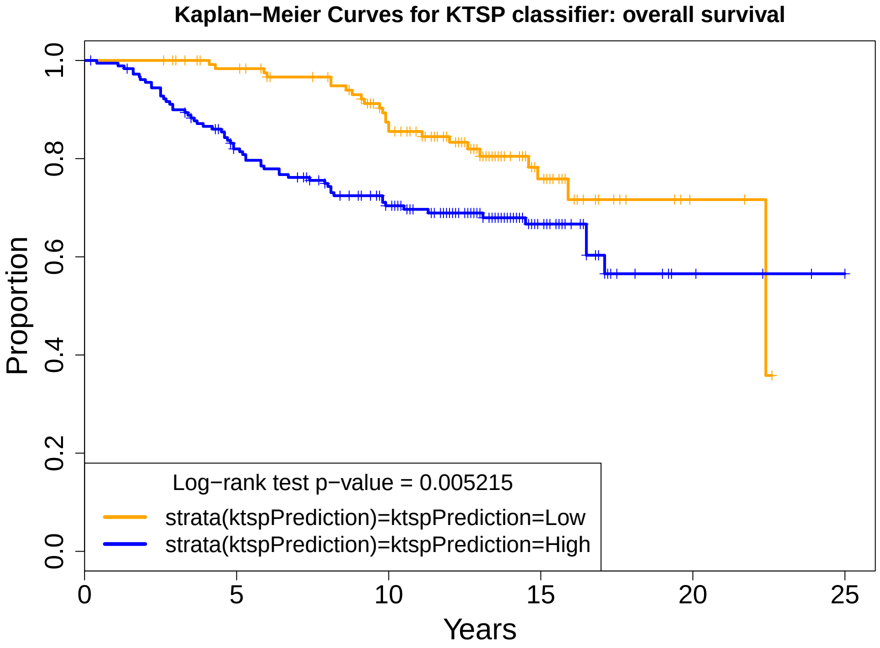

Figure 18: Kaplan-Meier curves for k-TSP prediction: overall survival.

The R code chunk below was used to compute the Kaplan-Meier curves for overall survival as stratified by the MammaPrint prediction.

```
> ###Kaplan-Meier curves stratified by MammaPrint
> mammaPrintKM.os <- survfit(survPH.os~strata(mammaPrintPredictionAll))
```

The R code chunk below was used to compute the univariate Cox proportional hazard model for overall survival

ad stratified by the MammaPrint prediction.

```
> ###Cox proportional hazard model with MammaPrint
> mammaPrintCOX.os <- coxph(survPH.os ~ mammaPrintPredictionAll)
```

Below the results of the univariate Cox proportional hazard model are shown:

```
> ###Cox proportional hazard model results
> summary(mammaPrintCOX.os)

Call:
coxph(formula = survPH.os ~ mammaPrintPredictionAll)

  n= 307, number of events= 82

                             coef exp(coef) se(coef)     z Pr(>|z|)
mammaPrintPredictionAllHigh 1.050     2.857    0.279 3.763 0.000168 ***
---
Signif. codes:  0 '***' 0.001 '**' 0.01 '*' 0.05 '.' 0.1 ' ' 1

                            exp(coef) exp(-coef) lower .95 upper .95
mammaPrintPredictionAllHigh     2.857     0.3501     1.653     4.935

Concordance= 0.624  (se = 0.029 )
Rsquare= 0.054   (max possible= 0.941 )
Likelihood ratio test= 17.06  on 1 df,   p=3.622e-05
Wald test            = 14.16  on 1 df,   p=0.0001682
Score (logrank) test = 15.5  on 1 df,   p=8.272e-05
```

In Figure 19 Kaplan-Meier curves for overall survival as tratified by the MammaPrint prediction.

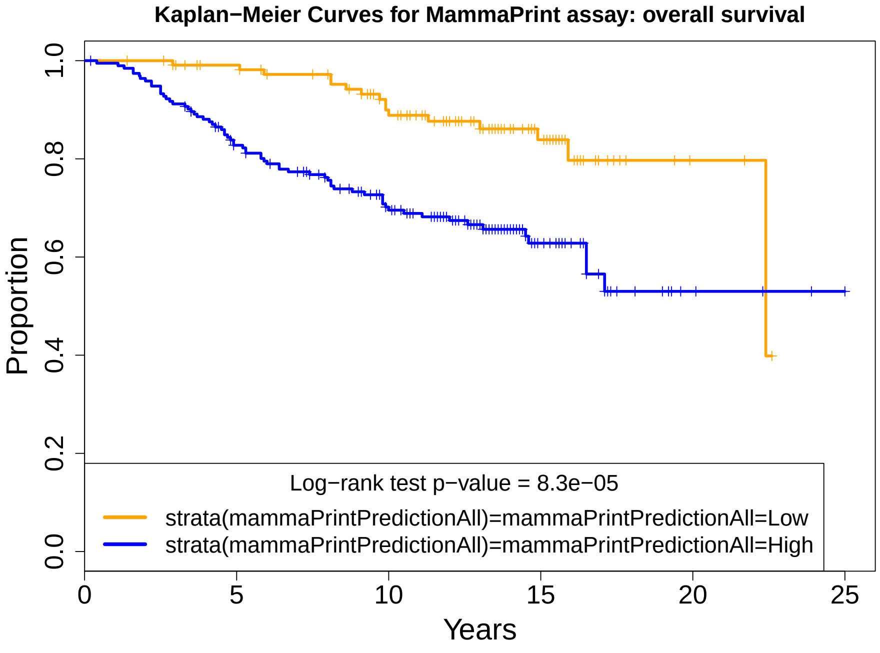

Figure 19: Kaplan-Meier curves for mammaPrint prediction: overall survival.

The R code chunk below was used to compute the Kaplan-Meier curves for overall survival as stratified by the LDA prediction.

```
> ###Kaplan-Meier curves stratified by LDA
> ldaKM.os <- survfit(survPH.os~strata(ldaPredictionAll))
```

The R code chunk below was used to compute the univariate Cox proportional hazard model for overall survival ad stratified by the LDA prediction.

```
> ###Cox proportional hazard model with LDA
> ldaCOX.os <- coxph(survPH.os ~ ldaPredictionAll)
```

Below the results of the univariate Cox proportional hazard model are shown:

```
> ###Cox proportional hazard model results
> summary(ldaCOX.os)

Call:
coxph(formula = survPH.os ~ ldaPredictionAll)

  n= 307, number of events= 82

                        coef exp(coef) se(coef)      z Pr(>|z|)
ldaPredictionAllHigh -0.1253    0.8822   0.2501 -0.501    0.616

                     exp(coef) exp(-coef) lower .95 upper .95
ldaPredictionAllHigh    0.8822      1.134    0.5404      1.44

Concordance= 0.519  (se = 0.025 )
Rsquare= 0.001   (max possible= 0.941 )
Likelihood ratio test= 0.25  on 1 df,   p=0.6197
Wald test            = 0.25  on 1 df,   p=0.6163
Score (logrank) test = 0.25  on 1 df,   p=0.616
```

In Figure 20 Kaplan-Meier curves for overall survival as tratified by the LDA prediction.

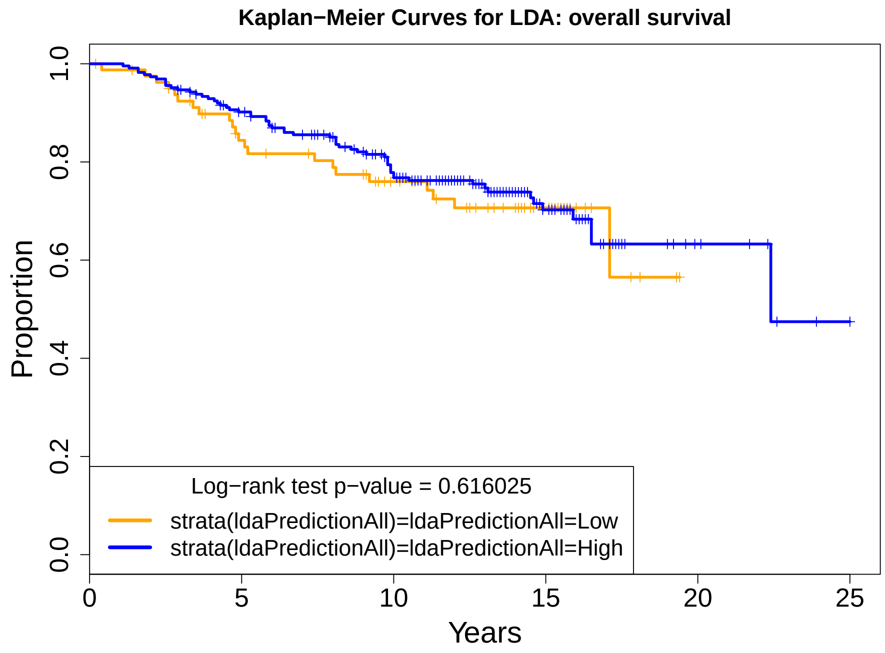

Figure 20: Kaplan-Meier curves for LDA prediction: overall survival.

The R code chunk below was used to compute the Kaplan-Meier curves for overall survival as stratified by the PAM prediction.

```
> ###Kaplan-Meier curves stratified by PAM
> pamKM.os <- survfit(survPH.os~strata(pamPredictionAll))
```

The R code chunk below was used to compute the univariate Cox proportional hazard model for overall survival ad stratified by the PAM prediction.

```
> ###Cox proportional hazard model with PAM
> pamCOX.os <- coxph(survPH.os ~ pamPredictionAll)
```

Below the results of the univariate Cox proportional hazard model are shown:

```
> ###Cox proportional hazard model results
> summary(pamCOX.os)

Call:
coxph(formula = survPH.os ~ pamPredictionAll)

  n= 307, number of events= 82

                      coef exp(coef) se(coef)     z Pr(>|z|)
pamPredictionAllHigh 0.7489    2.1147   0.2268 3.303 0.000958 ***
---
Signif. codes:  0 '***' 0.001 '**' 0.01 '*' 0.05 '.' 0.1 ' ' 1

                     exp(coef) exp(-coef) lower .95 upper .95
pamPredictionAllHigh     2.115     0.4729     1.356     3.298

Concordance= 0.63  (se = 0.029 )
Rsquare= 0.036   (max possible= 0.941 )
Likelihood ratio test= 11.26  on 1 df,   p=0.0007913
Wald test            = 10.91  on 1 df,   p=0.0009579
Score (logrank) test = 11.42  on 1 df,   p=0.0007261
```

In Figure 21 Kaplan-Meier curves for overall survival as tratified by the PAM prediction.

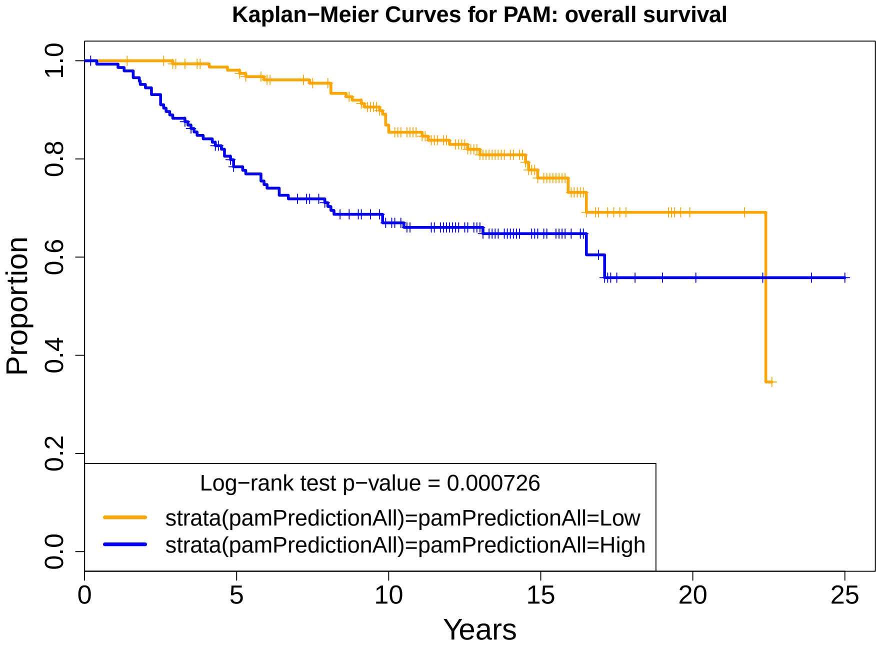

Figure 21: Kaplan-Meier curves for PAM prediction: overall survival.

5.6.4 Summary of Cox proportional hazards models analysis

Table 11 summarizes the results from univariate Cox proportional hazard models.

Table 11: Cox proportional hazard model results

Model	Hazard.Ratio	Confidence.Interval	P.value
ktsp prediction for DFS	1.43	1.01 - 2.02	0.039429
ktsp prediction for OS	1.95	1.21 - 3.14	0.00437
ktsp prediction for TTM	1.72	1.07 - 2.77	0.022707
lda prediction for DFS	0.89	0.61 - 1.30	0.548052
lda prediction for OS	0.88	0.54 - 1.44	0.619666
lda prediction for TTM	0.93	0.56 - 1.55	0.772328
mammaPrint prediction for DFS	1.51	1.05 - 2.16	0.021675
mammaPrint prediction for OS	2.86	1.65 - 4.94	3.6e-05
mammaPrint prediction for TTM	2.26	1.33 - 3.84	0.001244
pam prediction for DFS	1.34	0.96 - 1.87	0.082696
pam prediction for OS	2.11	1.36 - 3.30	0.000791
pam prediction for TTM	1.87	1.19 - 2.95	0.006268

5.7 Stratification by estrogen receptor status

Below are shown the classification results for the MammaPrint test and the K-TSP classifier stratified by Estrogen Receptor status as reported for the Buyse cohort (ER-positive and ER-negative).

```
> ###Format ER status
> ERstatus <- factor(pBuyse[,"Factor.Value.ER.status"])
> levels(ERstatus) <- gsub("Estrogen Receptor ", "", levels(ERstatus))
> levels(ERstatus)[levels(ERstatus) == "unknown"] <- NA
```

```
> ###Ktsp + MammaPrint + ER
> table(ktspPrediction, mammaPrintPredictionAll, ERstatus)
, , ERstatus = Negative

              mammaPrintPredictionAll
ktspPrediction Low High
          Low    3    6
          High   2   79

, , ERstatus = Positive

              mammaPrintPredictionAll
ktspPrediction Low High
          Low   88   25
          High  18   81
```

6 System information

Session information:

```
> toLatex(sessionInfo())
```

- R Under development (unstable) (2013-02-11 r61902), `x86_64-apple-darwin10.8.0`
- Locale: `C`
- Base packages: base, datasets, grDevices, graphics, grid, methods, parallel, splines, stats, utils
- Other packages: AnnotationDbi 1.21.16, Biobase 2.19.3, BiocGenerics 0.5.6, BiocInstaller 1.9.8, DBI 0.2-5, KernSmooth 2.23-10, MASS 7.3-24, MLInterfaces 1.39.5, RSQLite 0.11.2, RWeka 0.4-16, Snowball 0.0-9, annotate 1.37.4, caTools 1.14, cacheSweave 0.6-1, caret 5.15-61, class 7.3-6, cluster 1.14.4, e1071 1.6-1, filehash 2.2-1, foreach 1.4.0, gdata 2.12.0, genefilter 1.41.4, gplots 2.11.0, gtools 2.7.1, impute 1.33.0, lattice 0.20-15, limma 3.15.18, lsa 0.63-3, mammaPrintData 0.99.5, org.Hs.eg.db 2.9.0, pROC 1.5.4, pamr 1.54, plyr 1.8, rda 1.0.2-2, reshape2 1.2.2, rpart 4.1-1, seventyGeneData 0.99.5, sfsmisc 1.0-23, stashR 0.3-5, survival 2.37-4, switchBox 0.99.3, xtable 1.7-1
- Loaded via a namespace (and not attached): IRanges 1.17.41, Matrix 1.0-12, RWekajars 3.7.9-1, XML 3.96-0.2, bitops 1.0-5, codetools 0.2-8, digest 0.6.3, iterators 1.0.6, mboost 2.2-2, rJava 0.9-4, stats4 3.0.0, stringr 0.6.2, tools 3.0.0

7 References

References

[1] van 't Veer LJ, Dai H, van de Vijver MJ, He YD, Hart AAM, Mao M, Peterse HL, van der Kooy K, Marton MJ, Witteveen AT, Schreiber GJ, Kerkhoven RM, Roberts C, Linsley PS, Bernards R, Friend SH: **Gene expression profiling predicts clinical outcome of breast cancer**. *Nature* 2002, **415**(6871):530–6.

[2] van de Vijver MJ, He YD, van't Veer LJ, Dai H, Hart AA, Voskuil DW, Schreiber GJ, Peterse JL, Roberts C, Marton MJ, Parrish M, Atsma D, Witteveen A, Glas A, Delahaye L, van der Velde T, Bartelink H, Rodenhuis S, Rutgers ET, Friend SH, Bernards R: **A gene-expression signature as a predictor of survival in breast cancer**. *N Engl J Med* 2002, **347**(25):1999–2009. [1533-4406 (Electronic) Evaluation Studies Journal Article].

[3] Glas AM, Floore A, Delahaye LJ, Witteveen AT, Pover RC, Bakx N, Lahti-Domenici JS, Bruinsma TJ, Warmoes MO, Bernards R, Wessels LF, Van't Veer LJ: **Converting a breast cancer microarray signature into a high-throughput diagnostic test**. *BMC Genomics* 2006, **7**:278, [http://www.ncbi.nlm.nih.gov/entrez/query.fcgi?cmd=Retrieve&db=PubMed&dopt=Citation&list_uids=17074082]. [1471-2164 (Electronic) Journal Article Research Support, Non-U.S. Gov't].

[4] Buyse M, Loi S, van't Veer L, Viale G, Delorenzi M, Glas AM, d'Assignies MS, Bergh J, Lidereau R, Ellis P, Harris A, Bogaerts J, Therasse P, Floore A, Amakrane M, Piette F, Rutgers E, Sotiriou C, Cardoso F, Piccart MJ: **Validation and clinical utility of a 70-gene prognostic signature for women with node-negative breast cancer**. *J Natl Cancer Inst* 2006, **98**(17):1183–92, [http://www.ncbi.nlm.nih.gov/entrez/query.fcgi?cmd=Retrieve&db=PubMed&dopt=Citation&list_uids=16954471]. [1460-2105 (Electronic) Journal Article Multicenter Study Research Support, Non-U.S. Gov't Validation Studies].

[5] Geman D, d'Avignon C, Naiman DQ, Winslow RL: **Classifying gene expression profiles from pairwise mRNA comparisons.** *Stat Appl Genet Mol Biol* 2004, **3**:Article19, [http://dx.doi.org/10.2202/1544-6115.1071].

[6] Leek JT: **The tspair package for finding top scoring pair classifiers in R.** *Bioinformatics* 2009, **25**(9):1203–1204, [http://dx.doi.org/10.1093/bioinformatics/btp126].

[7] Peng RD: **Reproducible research in computational science.** *Science* 2011, **334**(6060):1226–1227, [http://dx.doi.org/10.1126/science.1213847].

[8] Brazma A, Parkinson H, Sarkans U, Shojatalab M, Vilo J, Abeygunawardena N, Holloway E, Kapushesky M, Kemmeren P, Lara GG, Oezcimen A, Rocca-Serra P, Sansone SA: **ArrayExpress–a public repository for microarray gene expression data at the EBI**. *Nucleic Acids Res* 2003, **31**:68–71. [1362-4962 (Electronic) Journal Article].

[9] Marchionni L, Wilson RF, Marinopoulos SS, Wolff AC, Parmigiani G, Bass EB, Goodman SN: **Impact of gene expression profiling tests on breast cancer outcomes.** *Evid Rep Technol Assess (Full Rep)* 2007, (160):1–105.

[10] Marchionni L, Wilson RF, Wolff AC, Marinopoulos S, Parmigiani G, Bass EB, Goodman SN: **Systematic review: gene expression profiling assays in early-stage breast cancer.** *Ann Intern Med* 2008, **148**(5):358–369.

[11] Troyanskaya O, Cantor M, Sherlock G, Brown P, Hastie T, Tibshirani R, Botstein D, Altman RB: **Missing value estimation methods for DNA microarrays.** *Bioinformatics* 2001, **17**(6):520–525.

[12] Tibshirani R, Hastie T, Narasimhan B, Chu G: **Diagnosis of multiple cancer types by shrunken centroids of gene expression**. *Proc Natl Acad Sci U S A* 2002, **99**(10):6567–72.

[13] Robin X, Turck N, Hainard A, Tiberti N, Lisacek F, Sanchez JC, Müller M: **pROC: an open-source package for R and S+ to analyze and compare ROC curves.** *BMC Bioinformatics* 2011, **12**:77, [http://dx.doi.org/10.1186/1471-2105-12-77].

[14] Weng L, Dai H, Zhan Y, He Y, Stepaniants SB, Bassett DE: **Rosetta error model for gene expression analysis.** *Bioinformatics* 2006, **22**(9):1111–1121, [http://dx.doi.org/10.1093/bioinformatics/btl045].

[15] Ravdin PM, Siminoff LA, Davis GJ, Mercer MB, Hewlett J, Gerson N, Parker HL: **Computer program to assist in making decisions about adjuvant therapy for women with early breast cancer.** *J Clin Oncol* 2001, **19**(4):980–991.
